# Graph Theoretical Characteristics of EEG-Based Functional Brain Networks in Patients With Epilepsy: The Effect of Reference Choice and Volume Conduction

**DOI:** 10.3389/fnins.2019.00221

**Published:** 2019-03-20

**Authors:** Maria N. Anastasiadou, Manolis Christodoulakis, Eleftherios S. Papathanasiou, Savvas S. Papacostas, Avgis Hadjipapas, Georgios D. Mitsis

**Affiliations:** ^1^KIOS Research and Innovation Centre of Excellence, Faculty of Engineering, University of Cyprus, Nicosia, Cyprus; ^2^Laboratory of Clinical Neurophysiology, Clinic B, Cyprus Institute of Neurology and Genetics, Nicosia, Cyprus; ^3^University of Nicosia Medical School, Nicosia, Cyprus; ^4^Department of Bioengineering, McGill University, Montreal, QC, Canada; ^5^Department of Electrical and Computer Engineering, KIOS Research Center, University of Cyprus, Nicosia, Cyprus

**Keywords:** epilepsy, volume conduction, montage, scalp EEG, graph theory, periodicities

## Abstract

It is well-established that both volume conduction and the choice of recording reference (montage) affect the correlation measures obtained from scalp EEG, both in the time and frequency domains. As a result, a number of correlation measures have been proposed aiming to reduce these effects. In our previous work, we have showed that scalp-EEG based functional brain networks in patients with epilepsy exhibit clear periodic patterns at different time scales and that these patterns are strongly correlated to seizure onset, particularly at shorter time scales (around 3 and 5 h), which has important clinical implications. In the present work, we use the same long-duration clinical scalp EEG data (multiple days) to investigate the extent to which the aforementioned results are affected by the choice of reference choice and correlation measure, by considering several widely used montages as well as correlation metrics that are differentially sensitive to the effects of volume conduction. Specifically, we compare two standard and commonly used linear correlation measures, cross-correlation in the time domain, and coherence in the frequency domain, with measures that account for zero-lag correlations: corrected cross-correlation, imaginary coherence, phase lag index, and weighted phase lag index. We show that the graphs constructed with corrected cross-correlation and WPLI are more stable across different choices of reference. Also, we demonstrate that all the examined correlation measures revealed similar periodic patterns in the obtained graph measures when the bipolar and common reference (Cz) montage were used. This includes circadian-related periodicities (e.g., a clear increase in connectivity during sleep periods as compared to awake periods), as well as periodicities at shorter time scales (around 3 and 5 h). On the other hand, these results were affected to a large degree when the average reference montage was used in combination with standard cross-correlation, coherence, imaginary coherence, and PLI, which is likely due to the low number of electrodes and inadequate electrode coverage of the scalp. Finally, we demonstrate that the correlation between seizure onset and the brain network periodicities is preserved when corrected cross-correlation and WPLI were used for all the examined montages. This suggests that, even in the standard clinical setting of EEG recording in epilepsy where only a limited number of scalp EEG measurements are available, graph-theoretic quantification of periodic patterns using appropriate montage, and correlation measures corrected for volume conduction provides useful insights into seizure onset.

## Introduction

The effect of reference choice and volume conduction on correlation measures obtained from scalp EEG is well-established; it has been shown that it may considerably influence measures of correlation in the time and frequency domains (Nunez et al., 1997). Specifically, both may introduce artificial zero-lag correlations: in the former case, referencing may result in the instantaneous subtraction of common signal components from different electrode time-series while in the latter case, instantaneous propagation of currents generated at a discrete source through the volume of the (head) conductor occurs (Nunez and Srinivasan, 2006; Christodoulakis et al., 2014). In turn, this makes the interpretation of EEG-based functional brain network characteristics more difficult. A number of correlation measures that attempt to reduce this influence have been proposed, including corrected cross-correlation, reduced/ imaginary coherence as well as standard, and weighted phase lag index, which by construction are insensitive to instantaneous (zero-lag) correlations, and are in principle less susceptible to volume conduction and reference effects (Nunez et al., 1997, 1999; Guevara et al., 2005; Marzetti et al., 2007; Stam et al., 2007; Haufe et al., 2010; Vinck et al., 2011; Nevado et al., 2012; Peraza et al., 2012; Thatcher, 2012; Christodoulakis et al., 2014; Chella et al., 2016). So far, the effects of reference choice and volume conduction on functional brain network properties have been assessed using short-duration EEG recordings (up to several minutes; Marzetti et al., 2007; Qin et al., 2010; Xu et al., 2014; Chella et al., 2016).

The properties of brain networks over longer time scales (multiple hours to days) have been investigated to a lesser degree; however, they may also convey important information about the underlying physiological and neural processes. Pronounced fluctuations have been revealed in the temporal evolution of global network characteristics (clustering coefficient, average shortest path length, assortativity) in patients with epilepsy (Kuhnert et al., 2010; Kramer et al., 2011; Geier et al., 2015; Anastasiadou et al., 2016). These fluctuations exhibit some periodic temporal structure which can be largely attributed to circadian rhythms. Furthermore, global properties of epileptic networks around seizure onset have been characterized using the clustering coefficient and shortest path length/efficiency (Lehnertz et al., 2014). The importance of local network properties (e.g., individual nodes) in epilepsy has also been explored (Kramer et al., 2008; Wilke et al., 2011; Varotto et al., 2012; Burns et al., 2014; Zubler et al., 2014; Geier et al., 2015).

Using a unique dataset of long-term (days) continuous scalp EEG recordings in patients with epilepsy, we recently showed that the summative properties (degree, efficiency, clustering coefficient) and topology of the resulting functional brain networks exhibit robust long-term periodicities in addition to the well-known circadian 24 h period (Anastasiadou et al., 2016; Mitsis et al., 2018). Our results demonstrated that brain network periodicities (particularly around 3 and 5 h) are strongly correlated to seizure onset. Furthermore, we showed that the modulation of brain network properties by the seizure events were relatively minor compared to these concurrent long-term fluctuations of brain network properties. Collectively, these results have important implications for seizure pathophysiology and suggest the potential of quantifying the long-term properties of EEG–based brain functional networks and incorporating information regarding their correlations with seizure onset for achieving seizure detection and prediction with improved sensitivity and specificity. However, these results were obtained using the bipolar montage on the basis of our previous work (Christodoulakis et al., 2013) and the functional brain networks were constructed using a relatively limited set of correlation measures.

Therefore, in the current study, our main aim was to investigate the extent to which these key findings would be reproducible for different, commonly-used montage choices as well as when different correlation measures with varying sensitivity to volume conduction and zero-lag correlations are used to construct functional brain networks. Specifically, we considered the following three reference choices (montages): the common reference montage (Cz), the average reference montage and the bipolar montage. Signal correlation measures are affected by the choice of reference and also by volume conducted currents from common sources (Nunez et al., 1997; Stam et al., 2007). We also showed that the choice reference and correlation measures influence the properties of the resulting functional brain networks around seizure onset (Christodoulakis et al., 2014). Thus, we considered the following correlation measures: cross-correlation (CC) in the time domain and coherence (COH) in the frequency domain, as well as measures that account for volume conduction effects and zero-lag (instantaneous) correlations: corrected cross-correlation (corCC), imaginary coherence (IC), standard phase lag index (PLI), and weighted phase lag index (WPLI) (Nunez et al., 1997, 1999; Guevara et al., 2005; Stam et al., 2007; Haufe et al., 2010; Vinck et al., 2011; Nevado et al., 2012; Peraza et al., 2012; Thatcher, 2012; Christodoulakis et al., 2014). In order to construct brain networks, we used long duration scalp EEG recordings (ranging between 21 and 94 h; 23 channels) in patients with epilepsy using all possible combinations between the above reference and correlation measure choices.

To our knowledge, this is the first study that investigates the influence of reference choice on the long-term periodic variations of scalp EEG-based functional brain networks. Furthermore, identifying the optimal combination between reference choice and correlation measure in the context of quantifying correlations between network properties and seizure onset is important as it may contribute to the design of improved detection/prediction algorithms which can take into account periodic variations in the state of the underlying functional brain networks.

## EEG Recordings and Preprocessing

Long-term video-EEG recordings were collected from nine patients with epilepsy and one patient with psychogenic seizures in the Neurology Ward of the Cyprus Institute of Neurology and Genetics. The study was approved by the Cyprus National Bioethics Committee. All subjects gave written informed consent in accordance with the Declaration of Helsinki. Six patients were monitored using the an XLTek (Natus Medical Incorporated, CA, USA) scalp EEG recording system (Patients 1–6), while the remaining four were monitored with the Nicolet (Natus Medical Incorporated, CA, USA) system (Patients 7–10). Table 1 summarizes the duration of the recordings, as well as the number and type of seizures of each patient. Seizures and sleep intervals were identified and marked by specialist neurophysiologists (coauthors ESP and SSP).

**Table 1:** EEG recordings.

**Patient**	**Length of recordings (h)**	**Number of seizures**	**Type of seizures**
1	46	1	Focal
2	22	2	Focal
3	68	2	Focal
4	94	1	Generalized
5	36	1	Generalized
6	24	0	Psychogenic
7	21	1	Focal
8	71	2	Focal
9	27	6	Generalized
10	69	4	Focal

Twenty-one electrodes were placed according to the 10–20 international system with two additional anterotemporal electrodes. In addition, four electrodes were used to record electrooculogram (EOG) and electrocardiogram (ECG) signals, respectively. The data were recorded at a sampling rate of 200 and 500 Hz for the XLTek and Nicolet systems, respectively. The EEG and EOG signals were band-pass filtered between 1 and 45 Hz to remove line noise and muscle artifacts. Next, we applied Lagged Auto-Mutual Information Clustering (LAMIC) (Nicolaou and Nasuto, 2007), using simultaneous EOG recordings to remove ocular artifacts.

It has been demonstrated that the montage (i.e., the choice of reference) affects correlation measures (Nunez et al., 1997) and as a consequence it may affect the corresponding graph-theoretic measures. For this reason, we mathematically converted the input data, which were originally recorded relative to the common cephalic reference, to three different montages: the common reference (Cz), the average reference and the bipolar montage (see section Recording Montages). We obtained results employing all three montages.

### Recording Montages

Scalp EEG recording devices use differential amplifiers in order to compute the voltage of each EEG channel. A differential amplifier takes as input the measurements of two electrodes and produces the corresponding EEG channel as the difference between the two inputs, after it has been amplified. The choice of input electrodes to each amplifier is known as montage.

The recordings obtained with our system is an example of common reference (Cz) where each amplifier takes as input one of the 10–20 system electrodes (Fp1, Fp2, F7, F3, Fz, F4, F8, T3, C3, Cz, C4, T4, T5, P3, Pz, P4, T6, O1, O2, A1, A2) and one reference electrode (REF) which is common to all amplifiers. This is an example of common reference (Cz) montage and we mathematically re-referenced the data to Cz, which is often the reference electrode of choice. The average reference montage subtracts the average signal over all channels (in our case, 19 scalp channels) or a carefully chosen subset of them from the signal at each channel. In this work, we used all 19 scalp channels to compute the average.

On the other hand, in the case of bipolar montage there is no input common to all the time-series but pairs of electrodes in nearby locations of the scalp are used to obtain the time-series by subtracting the corresponding measurements. Specifically, electrodes are taken in straight lines from the front to the back of the head, forming the pairs Fp1-F7, F7-T3, T3-T5, T5-O1, Fp2-F8, F8-T4, T4-T6, T6-O2, Fp1-F3, F3-C3, C3-P3, P3-O1, Fp2-F4, F4-C4, C4-P4, P4-O2, Fz-Cz, Cz-Pz.

### Functional Brain Network Construction

After obtaining the artifact-free time series, we calculated pairwise correlation measures between all pairs of time series (EEG data converted to common reference (Cz), average reference and bipolar reference montages) using the correlation measures described in section Correlation Measures. Each time series in the common (Cz), average and bipolar montages corresponds to a node in the network, which does not change over time. The edges or connections between the nodes are then identified by computing correlation measures between the corresponding time series. Specifically, if the corresponding measure between each pair exceeded a pre-specified threshold, the value of which was dependent on the employed measure (section Correlation Measures), edges were added between node pairs. All the connections (edges) identified in this way form a binary graph, which we term the functional brain network. Common measures for estimating the correlation between pairs of time series include CC, COH, synchronization likelihood, Granger causality, directed coherence, mutual information, PLI, and many more; see, e.g. (Pereda et al., 2005) for a review. The related changes in the brain network over time were tracked by using 5-s non-overlapping windows and quantifying the correlation between all time-series pairs, using the following measures: CC, corCC, COH, IC, PLI, and WPLI.

#### Correlation Measures

##### Cross correlation

Cross-correlation (CC) measures the similarity of two series as a function of the displacement of one relative to the other (Christodoulakis et al., 2014). For any pair of time series, *x*(*t*), and *y*(*t*), the normalized cross-correlation is calculated as follows:

(1) $$\begin{array}{r} C_{xy}\left(\tau \right)=\;\frac{1}{n-\tau }\sum_{t=1}^{n-\tau }\left(\frac{x\left(t\right)}{\sigma_{x}}\right)\left(\frac{y\left(t+\tau \right)}{\sigma_{y}}\right) \end{array}$$

where σ_*x*_ and σ_*y*_ are the standard deviations of *x* and *y*, respectively. The normalized CC, *C*_*xy*_, was computed for a range of values for the lag τ: a range of [−100 100] ms was examined here. *C*_*xy*_ takes values between −1 and 1, with 1 indicating perfect linear positive correlation, −1 perfect linear negative correlation and 0 no correlation. The maximum of the absolute value of CC, *max*_τ_|*C*_*xy*_|, over the chosen range of τ values, was used to quantify the degree of correlation between the two signals within a given time window.

##### Corrected cross correlation

Corrected cross-correlation (corCC) is a measure that is used in the case of scalp EEG measurements as CC often attains its maximum value at zero lag and zero-lag correlations are largely due to volume conduction effects or reference choice. For instance, according to the common reference (Cz) montage, the same signal is subtracted from all other electrode time-series. In order to measure true interactions not occurring at zero lag, we calculated the corCC ${\bar{C}}_{xy}\left(\tau \right)$, which is a measure of the autocorrelation sequence asymmetry, as defined in Nevado et al. (2012), by subtracting the negative-lag part of *C*_*xy*_(τ) from its positive-lag counterpart (Nevado et al., 2012):

(2) $$\begin{array}{r} {\bar{C}}_{xy}\left(\tau \right)=\;C_{xy}\left(\tau \right)-C_{xy}\left(-\tau \right)\text{ for }\tau >0 \end{array}$$

Note that ${\bar{C}}_{xy}\left(\tau \right)$ provides a lower bound estimate of the nonzero-lag cross-correlations and is notably smaller than *C*_*xy*_. As in the case of CC, the maximum within the same range of time lags ([−100 100] ms) is taken as the measure of correlation.

##### Coherence

Coherency is a widely-used measure for characterizing linear dependence between a pair of stochastic processes, as well as a quantitative measure of their phase consistency and may be viewed as the equivalent measure of cross-correlation in the frequency domain. Coherence (COH - *k*_*xy*_(*f*)), defined as the squared magnitude of coherency, is employed as a measure of correlation in the frequency domain (Pereda et al., 2005), i.e.:

(3) $$\begin{array}{r} k_{xy}\left(f\right)=\;\frac{\left|\langle S_{xy}\left(f\right)\rangle \right|}{\sqrt{\left|\langle S_{xx}\left(f\right)||S_{yy}\left(f\right)\rangle \right|}} \end{array}$$

The value of *k*_*xy*_(*f*) ranges between 0 and 1, with 1 indicating perfect linear correlation and 0 no correlation between *x* and *y* at frequency *f* . COH is a function of frequency; therefore, we calculated the maximum (with respect to frequency) COH value within the following frequency bands: broadband (1–45 Hz), delta (1–4 Hz), theta (4–8 Hz), alpha (8–13 Hz), beta (13–30 Hz), and gamma (30–45 Hz) in order to quantify the correlation between pairs of signals.

##### Imaginary coherence

The imaginary part of coherency (imaginary coherence—IC), as shown in Nolte et al. (2004), is less sensitive to volume conduction compared to its real part. Therefore, it can be used as a correlation measure to construct EEG-based functional brain networks:

(4) $$\begin{array}{r} IC_{xy}\left(f\right)=\;Imag\left(\Gamma_{xy}\left(f\right)\right) \end{array}$$

Here, we used the maximum (with respect to frequency) absolute value of the IC for the broadband signal as well as within the aforementioned frequency bands to quantify the correlation between the two signals.

##### Phase lag index

The phase lag index (PLI) was introduced in Stam et al. (2007), aiming to obtain a measure that provides reliable estimates of phase synchronization between two signals and is insensitive to volume conduction. Here, the instantaneous phases were obtained by initially bandpass filtering the signals within the frequency bands defined above and subsequently using the Hilbert transform to obtain the phase of the corresponding analytic signal., Phase difference distribution (Δϕ) as an index of asymmetry between a given pair of channels, that were wrapped in the interval, can be obtained in the following way:

(5) $$\begin{array}{r} PLI_{xy}=|\langle sgn\left(\Delta \phi \left(\tau \right)\right)\rangle | \end{array}$$

PLI ranges between 0 and 1, with 0 indicating no correlation and 1 maximal correlation.

##### Weighted phase lag index

The weighted phase lag index (WPLI) was proposed in Vinck et al. (2011) as an improved measure of phase synchronization for electrophysiological signals in the presence of noise and volume conduction. PLI may be sensitive to both of these factors, mostly due to its discontinuity, as small perturbations may convert phase lags into leads and vice versa. In contrast with PLI, WPLI weights the contribution of the observed phase leads and lags by the magnitude of the imaginary component of the cross-spectrum (Vinck et al., 2011):

(6) $$\begin{array}{l} WPLI=\frac{\left|\langle Imag(S_{xy}\left(f\right))\rangle \right|}{\langle |Imag(S_{xy}\left(f\right))|\rangle }\\ \;=\frac{\left|\langle |Imag(S_{xy}\left(f\right))|\cdot sgn(Imag(S_{xy}\left(f\right))\rangle \right|}{\langle |Imag(S_{xy}\left(f\right))|\rangle } \end{array}$$

Similar to PLI, WPLI ranges between 0 and 1, with 0 indicating no correlation and 1 maximal correlation.

#### Network Binarization

We constructed binary (rather than weighted) networks, i.e., networks with connections only between strongly correlated nodes. To do so, a threshold was set, the value of which depends on the employed correlation measure. Edges with weight values larger than the specified threshold were included in the graph (weight: 1), while edges with values less than the threshold were removed (weight: 0). We examined various threshold values for all the considered correlation measures. In all cases, different values yielded very similar results in terms of the observed patterns in network properties, provided that the threshold value was not too high (e.g., close to one for CC)—which yields disconnected graphs -, or too low (close to zero)—which yields densely/fully connected graphs. Therefore, we selected threshold values between these two extremes. The threshold value determines the actual value of the graph theoretic measures, but did not affect our results otherwise, as we are interested in the variation of these measures over time and the resulting periodic patterns and not specifically in their absolute value. We also used the method of phase-randomized surrogate data to construct binary networks (Theiler et al., 1992); however, this approach yielded densely connected networks, i.e., degree values that were similar to those obtained by setting a fixed, low threshold value. Moreover, the temporal patterns of the network summative properties were found to be overall clearer when thresholding was used (Sections Network Binarization and Periodicities in Functional Brain Network Properties). Taking also into account that thresholding is much faster to implement, which is important for real-time seizure detection/prediction applications, we selected the thresholding method and we selected the threshold values so that similar average degree values were obtained for different correlation measures and montages. Specifically, we selected the following threshold values for the bipolar and common (Cz) reference; CC: 0.65, COH: 0.65, IC: 0.58, corCC: 0.1, PLI: 0.1, and WPLI: 0.45. For the average reference, the corresponding values were as follows; CC: 0.65, COH: 0.65, IC: 0.4, corCC: 0.1, PLI: 0.1, and WPLI: 0.45.

### Graph Theoretic Measures

For each subject, the evolution of the functional brain network over time was monitored by observing how different measures of the corresponding graph changed over time: average degree, global efficiency, and clustering coefficient.

#### Average Degree

The degree *k*_*i*_ of a node *i* is defined as the number of connections or edges that this node has with other neighboring nodes in the network. The average degree of a network is the average value of the summary of degrees of a network and quantifies how well-connected the corresponding graph is (Rubinov and Sporns, 2010):

(7) $$\begin{array}{r} K=\;\frac{1}{n}\;\sum_{i\epsilon N}k_{i} \end{array}$$

#### Global Efficiency

The shortest path length, *d*_*ij*_, between a pair of nodes *i* and *j* is defined as the minimum number of edges that have to be traversed to get from node *i* to *j*. The characteristic path length is defined as the average shortest path length over all pairs of nodes in the network and is a measure of how efficient the information flow through the network is (Christodoulakis et al., 2014):

(8) $$\begin{array}{r} L=\;\frac{1}{n\left(n-1\right)}\sum_{i,j\epsilon N,i\neq j}d_{ij} \end{array}$$

A limitation of the characteristic path length is that if any pair of nodes *i* and *j* is not connected through any path, the corresponding shortest path length value is *d*_*ij*_ = ∞ . Therefore, the characteristic path length is well-defined only for pairs of nodes that are connected. To overcome this limitation, efficiency between a pair of nodes was defined as the inverse of the shortest distance between the nodes, ${\;}^{1}{/}_{d_{ij}}$ (Latora and Marchiori, 2001):

(9) $$\begin{array}{r} E=\;\frac{1}{n\left(n-1\right)}\sum_{i,j\epsilon N,\;i\neq j}\frac{1}{d_{ij}} \end{array}$$

Global efficiency is defined as the average efficiency over all pairs of nodes (Latora and Marchiori, 2001).

#### Clustering Coefficient

A cluster in a graph is a group of nodes that is highly interconnected. The clustering coefficient *C*_*i*_ of a node *i* is defined as the fraction of existing edges between nodes adjacent to node *i*, over the maximum possible number of such edges (Watts and Strogatz, 1998).

(10) $$\begin{array}{r} C_{i}=\;\frac{2t_{i}}{k_{i\;}\left(k_{i}-1\right)} \end{array}$$

where *k*_*i*_ is the degree of node *i*, and *t*_*i*_ denotes the number of edges, $e_{jj^{\prime }}$, between pairs of nodes, *j* and *j*', that are both connected to *i*. Consequently, the clustering coefficient of the network *C* is defined as the mean clustering coefficient among all network nodes.

(11) $$\begin{array}{r} C=\;\frac{1}{n}\sum_{i\in N}C_{i} \end{array}$$

### Periodicity Estimation

One of our main aims was to characterize the periodicities that arise in functional brain network characteristics over a wide range of time scales. Each of the three network properties—average degree, global efficiency, and clustering coefficient, which were used for monitoring functional brain networks, provides a single value per network constructed from a 5-s window, thus forming a time series across the entire recording time. We utilized the Lomb-Scargle periodogram (Scargle, 1982) to obtain the power spectral density (PSD) of the time series for each network summative property (e.g., degree). This was done for all correlation measures and montages. The Lomb-Scargle periodogram is more appropriate for unevenly sampled data and in our case, we observed that in some patients we had small gaps in the signal measurements.

### Circular Statistics

To investigate the relation of seizure onset to brain network periodicities and the influence of reference choice effect on these periodicities, we calculated the instantaneous phase at seizure onsets for each periodic component and obtained the corresponding phase distribution. Subsequently, we used circular statistics to examine whether seizure onsets occurred at specific/preferred phases, as opposed to random phases. To this end, we first performed zero-phase digital filtering of the average degree time series to obtain band limited signals around the main identified peaks on a subject-to-subject basis (±0.5 h before and after the main peak of each periodic component). Specifically, we focused this analysis on the periodic components that were found to be consistent across patients. Significant peaks identified in individual subjects were deemed consistent if their location relative to the mean location across subjects was less or equal to half an hour. More details on the location of observed peaks are given in Section Periodicities in Functional Brain Network Properties.

Subsequently, we applied the Hilbert transform on the band limited signals to calculate the instantaneous phase of each periodic component (Klingspor, 2015). For all patients, the instantaneous phases at the onset of the seizure were collected and the obtained phase distribution were subsequently investigated using CircStat which is a Matlab (Math works, Natick MA) toolbox related to circular statistics (Berens, 2009). For more details, the reader is referred to Mitsis et al. (2018). To investigate whether phase values at seizure onset times were distributed uniformly around the circle from 0 to 2π, we applied the Rayleigh test with the null hypothesis (*H*_0_) being that the population is distributed uniformly around the circle. The Rayleigh test computes the resultant vector length *R* that suggests a non-uniform distribution (Fisher, 1993) and is particularly suited for detecting a unimodal deviation from uniformity. To account for the fact that we had multiple seizures for some subjects, we created groups of nine samples (one seizure per patient) for all possible combinations to obtain the corresponding corrected *p*-values (Zar, 1999). Note that for the 24 h periodic (circadian) component, these groups included six samples only, since the recordings of six out of ten patients were longer than 24 h.

## Results

The results related to the time-resolved functional brain measures correspond to Patient 4, since the longest recording (94 h) was obtained from this patient. Similar results were obtained in all ten patients (see also Supplementary Material).

### Network Binarization

**Figure 1:**
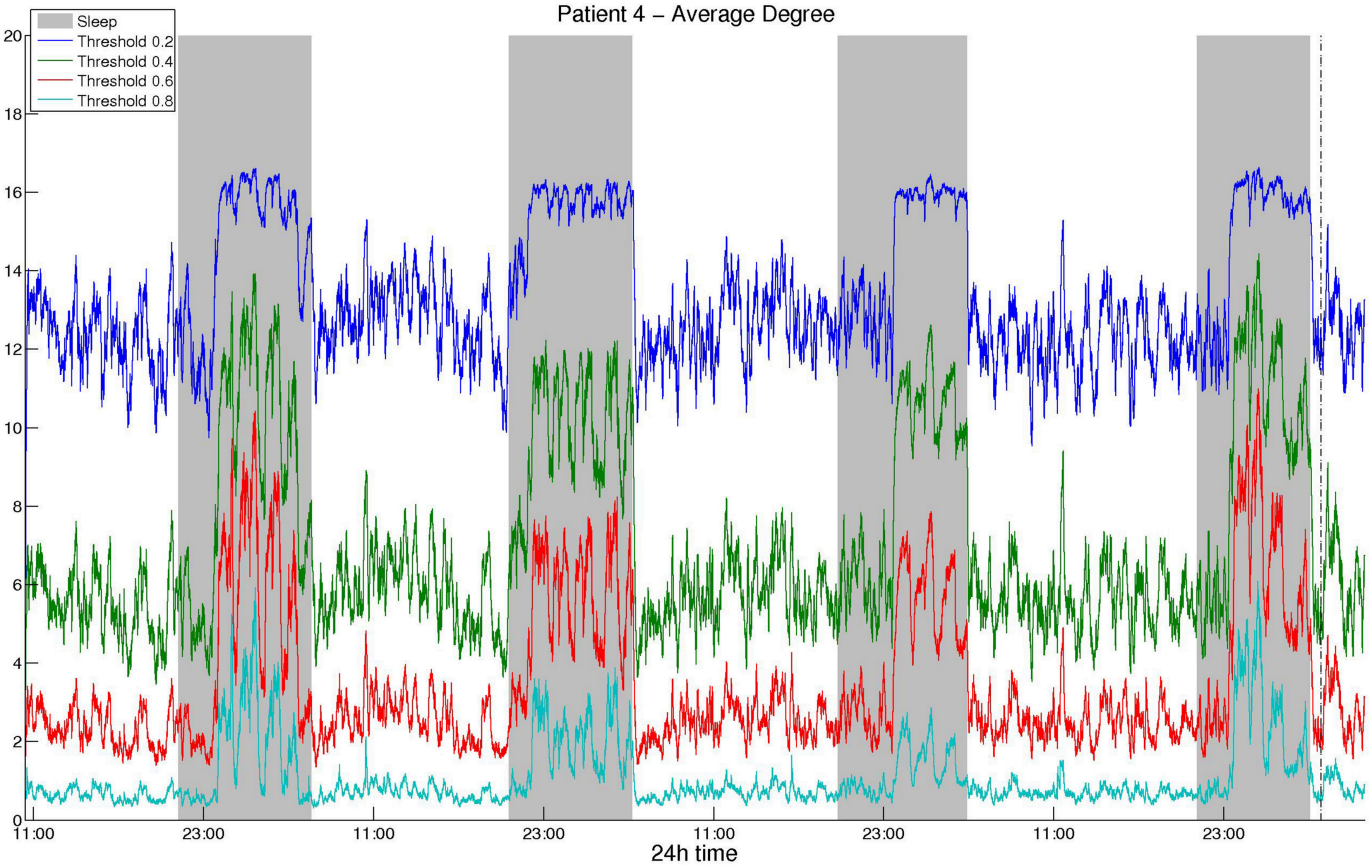
Smoothed average degree of the functional brain network of Patient 4, constructed using cross-correlation, for various thresholds. For all thresholds, the 24 h period is visible and the resulting patterns are similar, although details for some intermediate periodicities may be lost.

As mentioned above, we selected the thresholds aiming to avoid extreme values and obtain similar average degree values among different correlation measures and montages. Figure 1 illustrates the average network degree as obtained from CC using a bipolar montage for threshold values between 0.2 and 0.8, where it can be seen that the behavior for different threshold values is similar. It was found that the obtained network properties, e.g., average degree in this case, exhibit very similar patterns with respect to time, regardless of the threshold used. To provide comparisons with the surrogate data network binarization, we show the average degree obtained in the latter case in Figure S1 for CC, where it can be seen that the resulting degree values are high and that the circadian periodicity is not as clear as in Figure 1.

### Effect of Reference Choice on Network Measures and Their Periodicities

The choice of reference channel affects the local cortical estimates and their interactions with other locations. It is known that using a common reference (Cz) can substantially inflate coherence estimates, particularly at smaller distances (Nunez and Srinivasan, 2006) as a common signal is subtracted from all channels. On the other hand, the average reference is known to yield coherence estimates that are closer to coherence estimates obtained from reference-independent potentials (Nunez and Srinivasan, 2006). Note that the average reference is commonly used when a large number of electrodes with extensive coverage of the head is used. In our case, the standard 10–20 system was used, which may yield a poor approximation of the reference-free potentials (Nunez and Srinivasan, 2006; Christodoulakis et al., 2014). Furthermore, in the settings of a limited number of electrodes as in our case, the bipolar montage has been suggested for obtaining estimates of local superficial cortical generators (Nunez and Srinivasan, 2006; Christodoulakis et al., 2014).

Since the choice of montage influences the estimates of local activity (electrodes) and the interactions between them, it is also expected to influence the resulting graph theoretic measures and hence their periodic properties. We constructed functional networks using the correlation measures described in section Correlation Measures (CC, corCC, COH, IC, PLI, and WPLI) using long-term EEG data and all three montages [bipolar, common reference (Cz) and average reference]. Below, we study the effects of reference (montage) choice on the long-term properties of the resulting brain networks separately in the time and frequency domain, focusing on the emerging periodicities.

#### Time Domain

**Figure 2:**
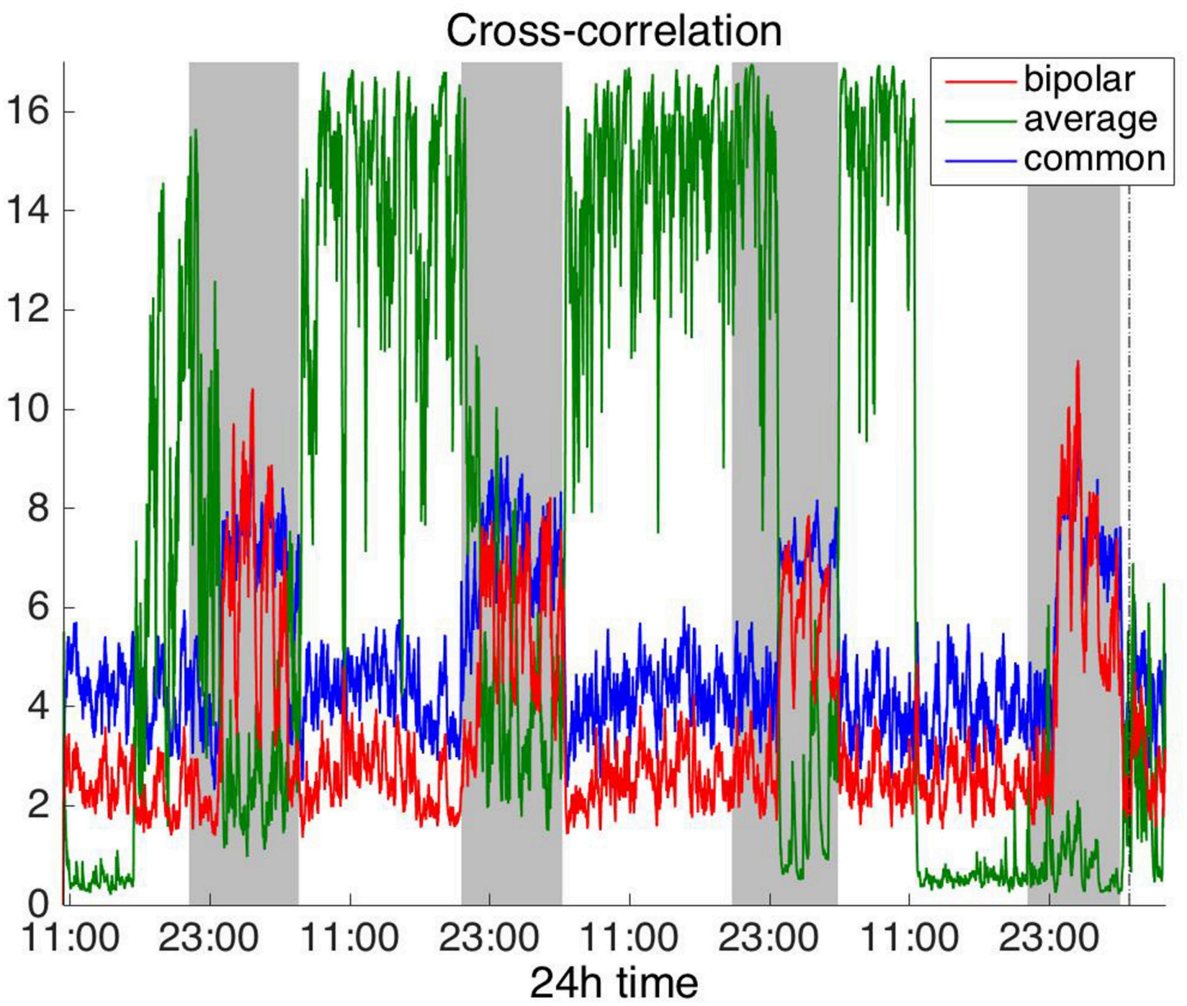
Average degree of the functional brain networks of Patient 4 as a function of time, using cross correlation for assessing pairwise correlations. The vertical dashed line indicates seizure onset and the gray bars indicate sleep intervals. Cross correlation yields an opposite 24 h periodic pattern when using the average reference. However, we can clearly observe a periodic pattern with a main period equal to around 24 h for all montages.

**Figure 3:**
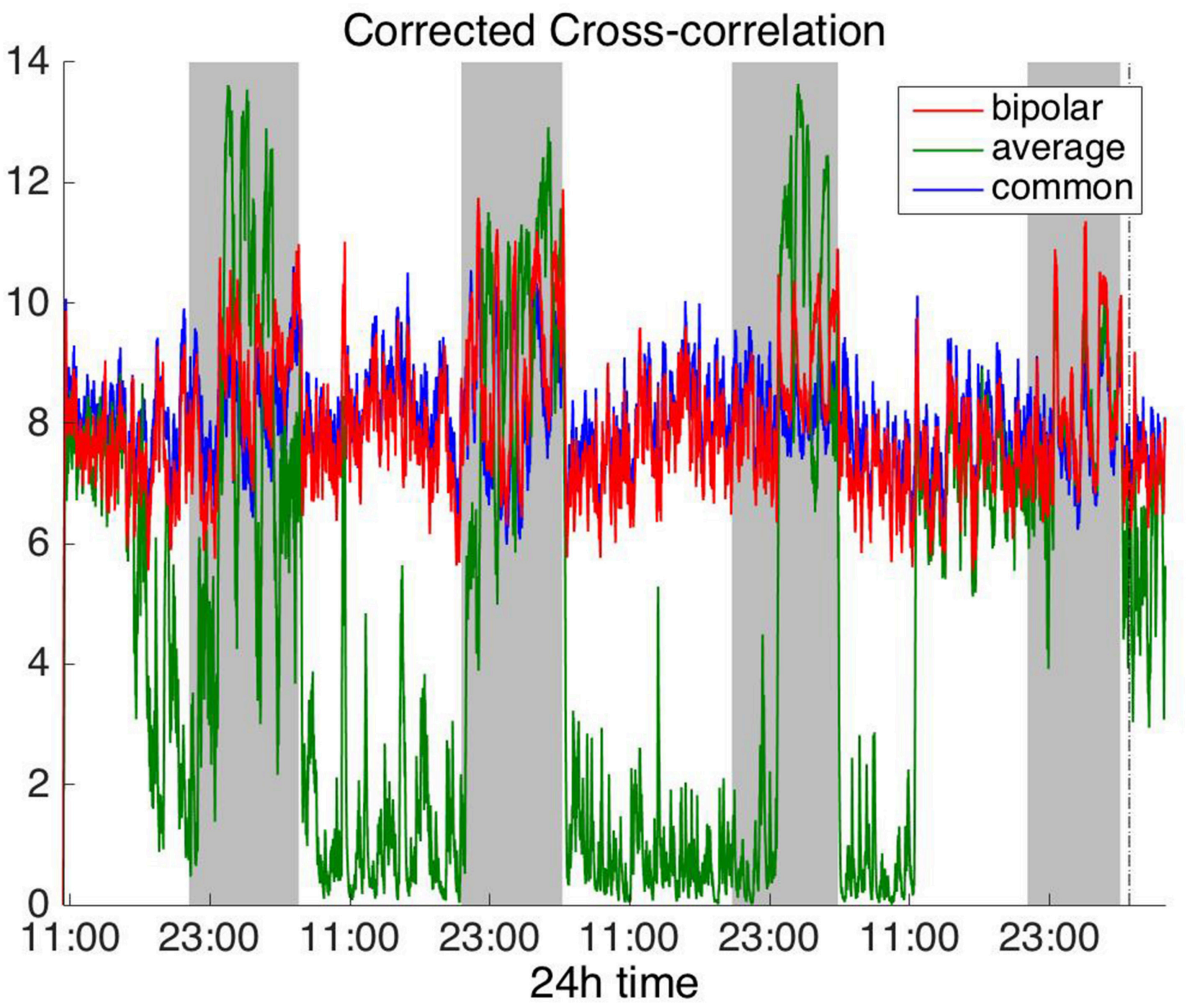
Average degree of the functional brain networks of Patient 4 as a function of time, using corrected cross correlation for assessing pairwise correlations. The vertical dashed line indicates seizure onset and the gray bars indicate sleep intervals. In contrast to cross correlation (Figure 2), corrected cross correlation yields the same 24 h periodic pattern for all montages.

Figures 2, 3 show the time course of the average network degree using CC (Figure 2) and corCC (Figure 3) for all montages. The green, red and blue lines correspond to average, bipolar, and common (Cz) reference, respectively. In Figure 2, it is evident that the functional brain networks yielded by CC are less connected (lower degree) during the time when the patient is awake compared to sleep (gray shaded bars) in the case of bipolar and common reference (Cz) montage. In contrast, the average montage yields the opposite trend. In Figure 3 it can be seen that, when corCC was used, the average reference yielded similar results to the other two montages (increased connectivity during sleep), suggesting that this measure is less susceptible to the choice of reference. In Figure S1, we can observe a similar 24 h periodic trend in the case of bipolar and common reference (Cz) montage for global network efficiency when CC was used. As before (Figure 2), the average reference montage yielded an opposite main periodic trend, which was however reversed when corCC was used (Figure S2). In Figures S3, S4, we show the clustering coefficient patterns obtained with CC and corCC, respectively for all reference montages, whereby similar observations can be made. Therefore, corCC yielded overall more consistent patterns for different montages for all three network measures. Along with the dominant 24 h cycles, additional periodic components at shorter time scales co-exist; note, for example, the spikes that occur at both awake- and sleep-times separated on average by ~75 min (e.g., Figures 2, 3). These weaker periodicities are examined in detail in section Periodicities in Functional Brain Network Properties.

#### Frequency Domain

**Figure 4:**
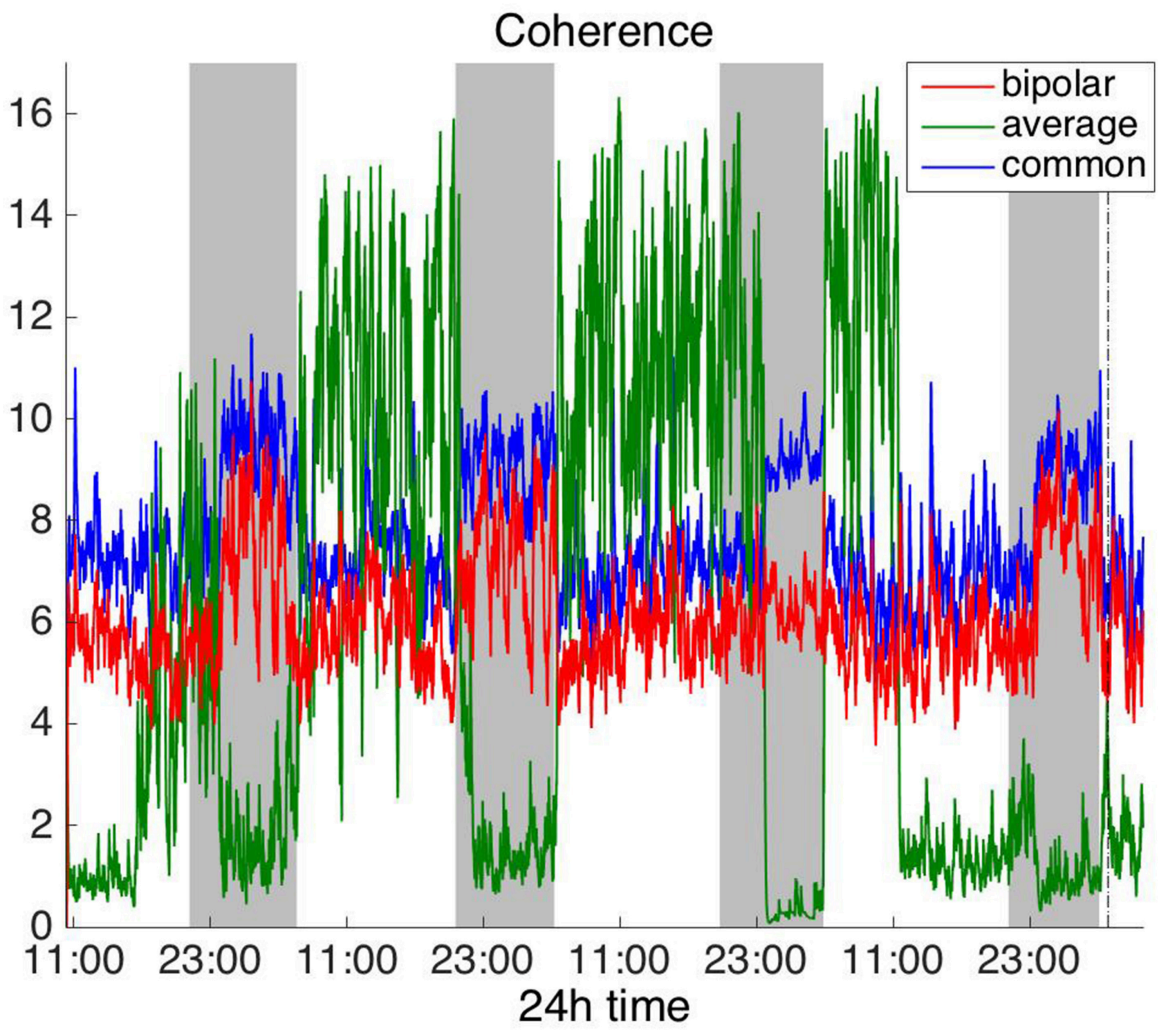
Average degree of the functional brain networks of Patient 4 as a function of time (broadband signal), using coherence for assessing pairwise correlations. The vertical dashed line indicates seizure onset and the gray bars indicate sleep intervals. Similarly to correlation (Figure 2), coherence yields an opposite 24 h periodic pattern when using the average reference.

**Figure 5:**
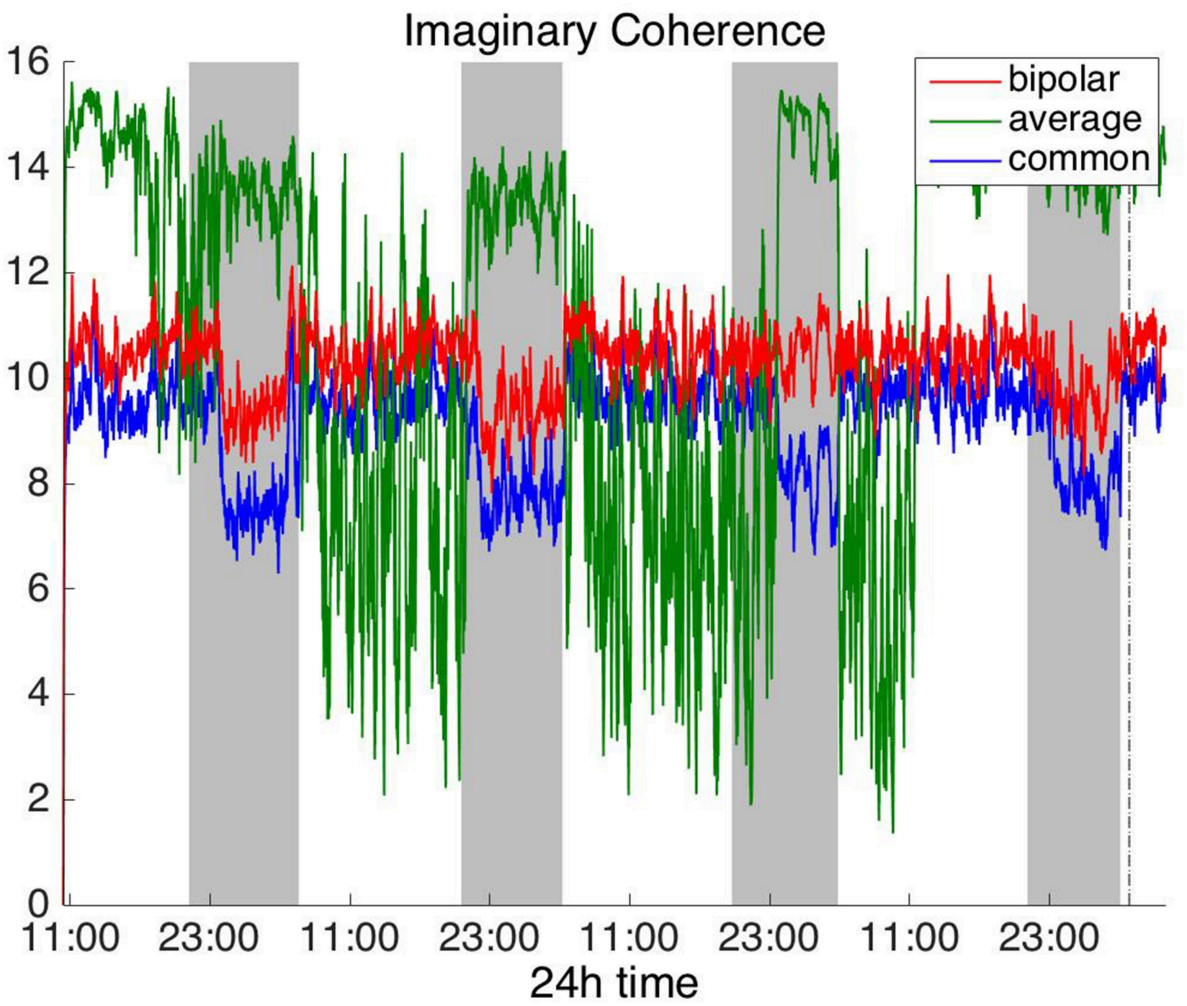
Average degree of the functional brain networks of Patient 4 as a function of time (broadband signal), using imaginary coherence for assessing pairwise correlations. The vertical dashed line indicates seizure onset and the gray bars indicate sleep intervals. Similarly to correlation (Figure 2) and coherence (Figure 4), imaginary coherence yields an opposite 24 h periodic pattern when using the average reference.

**Figure 6:**
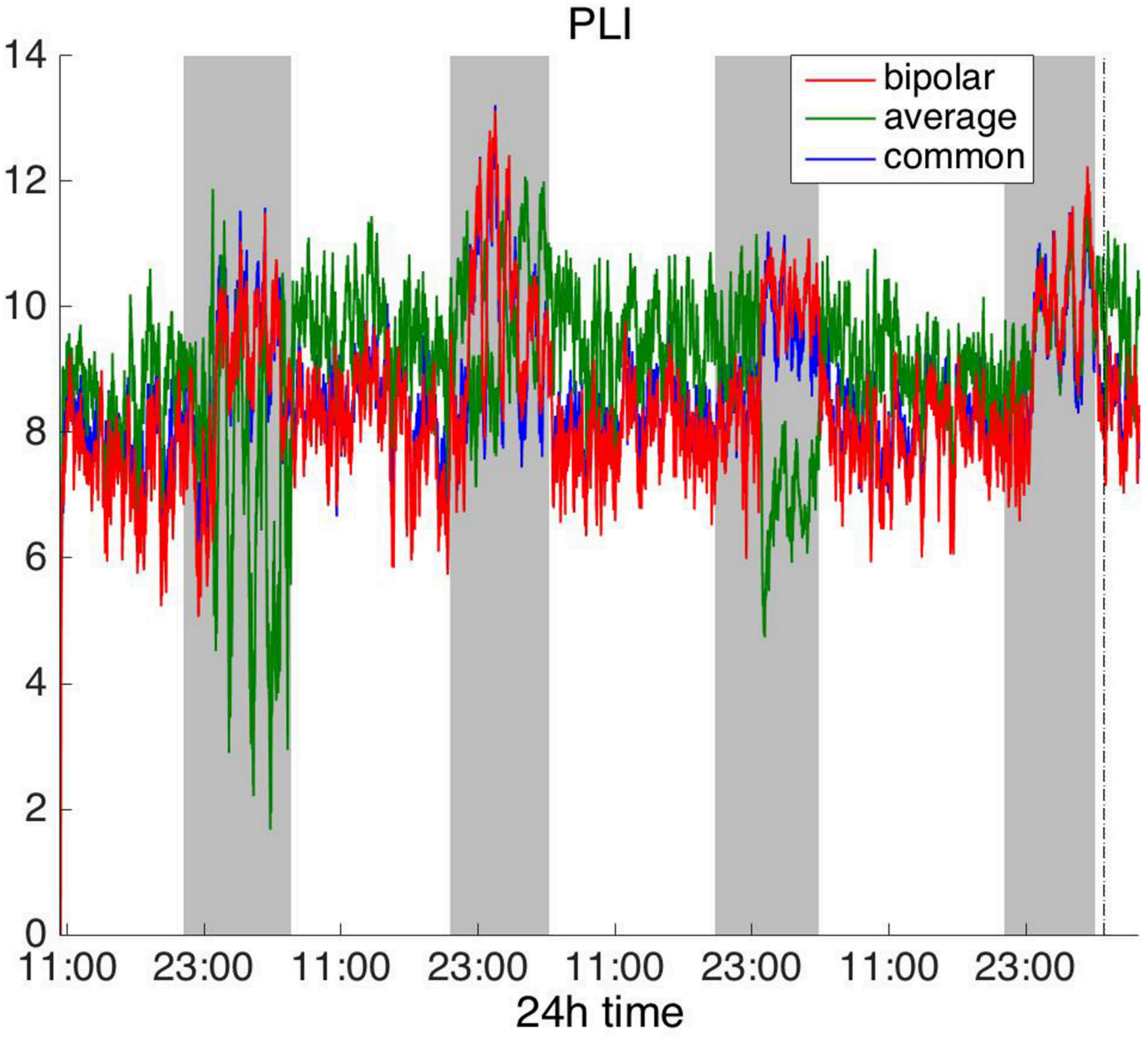
Average degree of the functional brain networks of Patient 4 as a function of time (broadband signal), using PLI for assessing pairwise correlations. The vertical dashed line indicates seizure onset and the gray bars indicate sleep intervals. PLI yields a different 24 h periodic pattern when using the average reference.

**Figure 7:**
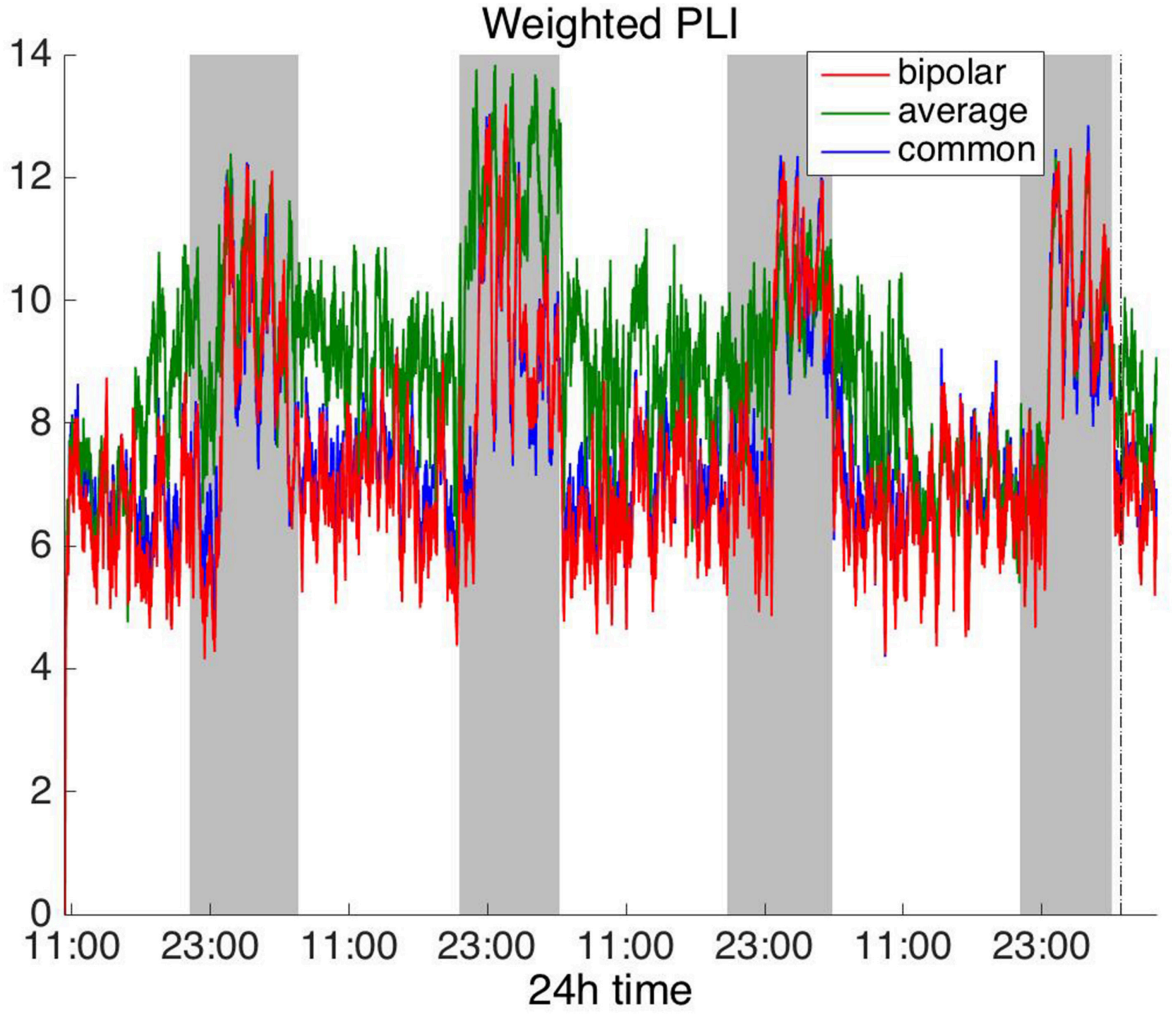
Average degree of the functional brain networks of Patient 4 as a function of time (broadband signal), using WPLI for assessing pairwise correlations. The vertical dashed line indicates seizure onset and the gray bars indicate sleep intervals. Similarly to corrected cross-correlation (Figure 3), WPLI yields the same 24 h periodic pattern for all montages.

To investigate brain network properties in the frequency domain, we constructed functional networks using COH, IC, PLI, and WPLI. Figures 4, 5 show the average degree for the broadband signal obtained from COH and IC for all montages, respectively. Figures 6, 7 show the average degree obtained from PLI and WPLI for all montages, respectively. Figures S5, S6 show the global efficiency and clustering coefficient, respectively when using WPLI for all montages. Note that the global efficiency and clustering coefficient are not shown separately for COH, IC, and PLI as they yielded similar periodic trends to the average degree for all montages. In Figure 4 (COH), we observe that the average reference montage yields opposite periodic trends compared to the other two montages, similarly to CC (section Time Domain). Also, we observed that COH within all frequency bands (delta, theta, alpha, beta, and gamma) yielded similar periodic trends to the broadband signal (Figure 4) for both the bipolar and common (Cz) montages (results not shown separately). In the case of the average reference montage, the trend observed for the broadband signal was mostly determined by the delta, theta and alpha band, which yielded opposite periodic trends to the beta and gamma bands. It was found that functional brain networks obtained with COH were less connected during the time when the patient was awake compared to sleep (gray shaded bars), in the case of bipolar, and common (Cz) reference montage.

In Figure 5 we can observe that IC exhibits a different behavior overall compared to COH and other correlation measures, with the bipolar and common (Cz) montages yielding opposite periodic trends. In contrast, the average reference montage yielded increased connectivity during sleep, exhibiting an opposite trend to the other two montages. The periodicities yielded by the broadband signal in the case of bipolar and common (Cz) montages were mostly determined by IC values in the beta and gamma bands and in the case of average reference montage they were mostly determined by IC values in the delta, theta and alpha bands, which yielded opposite periodic trends to the beta and gamma bands. The results suggest that IC and COH are influenced substantially by reference choice. As before, the main periodicity was the 24 h circadian cycle, which is evident in all the results.

In Figure 6, which shows the average degree obtained with the PLI for all montages, we can observe that the average reference yields opposite periodic trends compared to the other two montages. The functional brain networks using PLI are less connected during the time when the patient is awake compared to sleep in periodic cycles of ~24 h, in the case of the bipolar and common (Cz) reference montages. In Figure 7, we show that the average degree obtained using WPLI was less affected by reference choice compared to the rest of the correlation measures. The same can be observed in Figures S5, S6 for the efficiency and clustering coefficient, respectively. The WPLI-based functional brain networks were less connected during the time when the patient was awake compared to sleep for all montages. As before, along with the 24 h cycles, additional periodic components at shorter time scales co-exist in the time course of the average degree in the frequency domain (section Periodicities in Functional Brain Network Properties).

#### Periodicities in Functional Brain Network Properties

**Figure 8:**
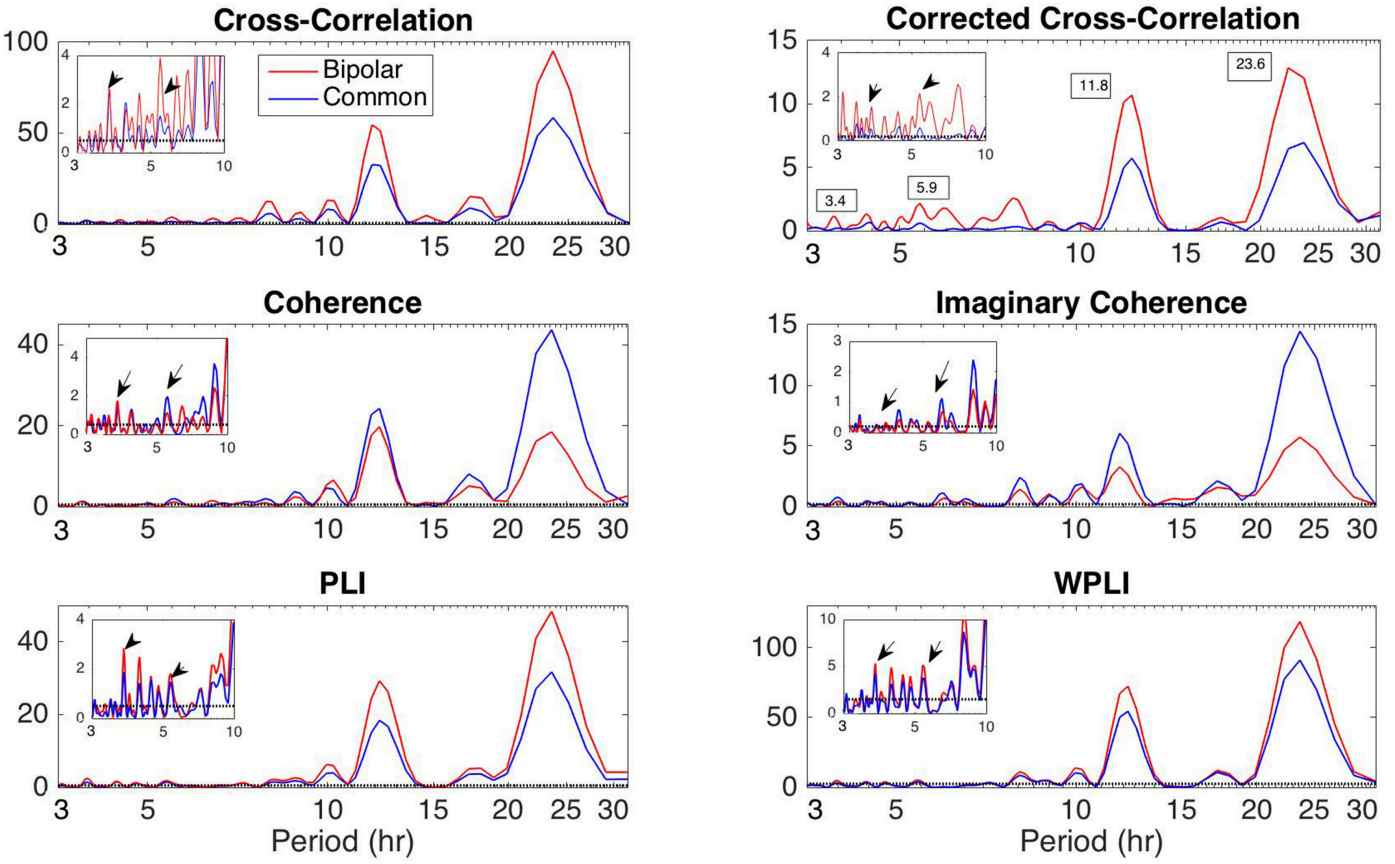
Lomb-Scargle periodogram of the average degree of the functional brain network of Patient 4 using cross-correlation, corrected cross-correlation, coherence, imaginary coherence, PLI, and WPLI for common reference (blue line) and bipolar montage (red line). The dotted horizontal lines denote the statistical significance level (*p* = 0.05). The arrows in the inset figures denote the periods around 3.6 and 5.4 h, which were identified across all subjects along with the peaks around 12 and 24 h. Corrected cross correlation and WPLI were affected less by reference choice.

Apart from the dominant 24 h periodicity in network properties, periodicities at smaller time scales can also be observed (Figures 2–7 and Figures S2–S7). In this section, we investigate these periodicities in more detail. Figure 8 illustrates the periodogram of the average degree of the functional brain networks of Patient 4 constructed using all correlation measures for the bipolar (red) and common (Cz; blue) reference. The horizontal lines denote the statistical significance level (*p* = 0.05), above which spectral peaks can be considered as significant. In Figure S8, we also show the Lomb-Scargle periodogram of the average degree obtained using surrogate data network binarization for the bipolar montage (Figure S1). Similar periodic components can be observed; however, the circadian periodicity is not as clear as in Figure 8. The average reference montage yielded substantially noisier results for all subjects and is shown in Figure S9; however, the main peaks (e.g., at 24 h) are evident in this case as well. The green, red and blue lines indicate the average, bipolar, and common (Cz) reference, respectively. The peaks in the periodogram correspond to periods of 3.4, 5.9, 11.8, and 23.6 h and have been marked accordingly in the case of corCC (Figure 8 and Figure S9). Similar peaks were observed for all other correlation measures. We observed four main peaks for all montages across patients, the location of which varied between 3.2 and 3.8 h (mean: 3.6 h), 4.9 and 5.9 h (mean: 5.4 h), 11.8 and 12.2 (mean:12 h), and 23.6 to 24.5 h (mean: 24 h). For simplicity, we refer to the main periodic peaks using these mean values from now on. The periodicity peaks at around 3.6, 5.4 h were observed in all subjects, while the peaks at around 12 and 24 h were identified in eight out of 10 subjects, and six out of six subjects, respectively (recall that recordings longer than 24 h were obtained for six subjects). In Figure S9 we can observe that, in the case of the average reference montage, the peaks are located at slightly different frequencies compared to the other two montages, especially in the case of CC, COH, and IC, indicating that these correlation measures are influenced by reference choice to larger extent. On the other hand, corCC and WPLI are affected less by reference choice and yield the most consistent frequency peaks overall. Figure S10 illustrates the Lomb-Scargle periodogram of the average degree of the functional brain networks obtained from Patient 6, who was the only psychogenic seizure patient in our cohort, constructed using all correlation measures for the bipolar (red) and common (Cz; blue) reference. The peaks in the periodogram correspond to periods of 3.4, 4.8, and 12.05 h and have been marked accordingly in the case of corCC (Figure S10). Note that the length of the recordings for this patient was 24 h; therefore, the peak at 24 h is not as clear as for subjects with longer recordings, except in the case of the corCC (Figure S10—top right panel).

### Circular Statistics

**Figure 9:**
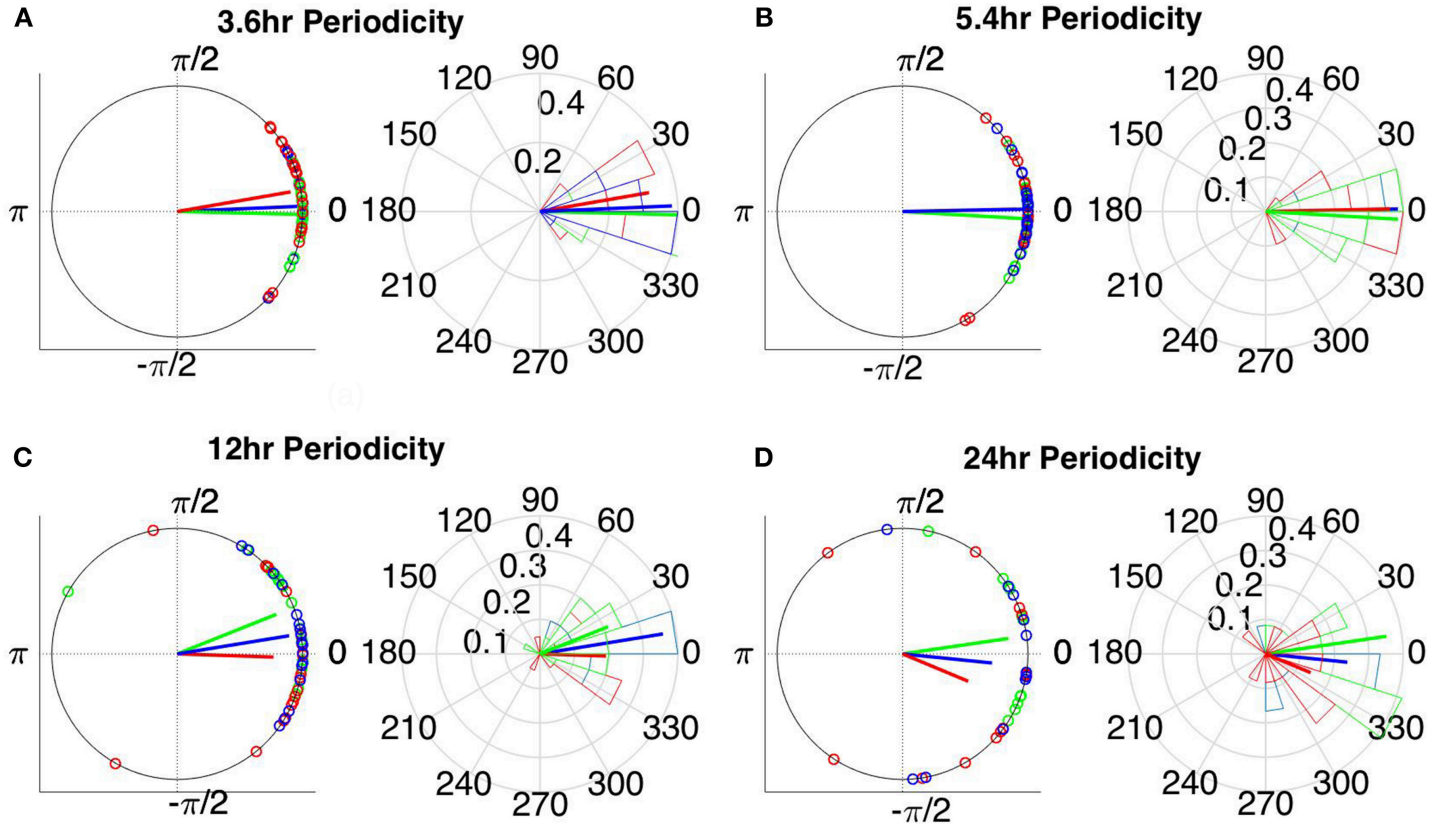
Instantaneous phases of the network average degree at seizure onset for the **(A)** 3.6 h, **(B)** 5.4 h, **(C)** 12 h, and **(D)** 24 h periodicities of all patients for corrected cross correlation. The left panels present the unit circle and the phases as points for all seizures from all patients. The right panels show the angular histogram of the distribution as well as the corresponding probability values. Blue, common reference (Cz); green, average reference; red, bipolar reference.

**Figure 10:**
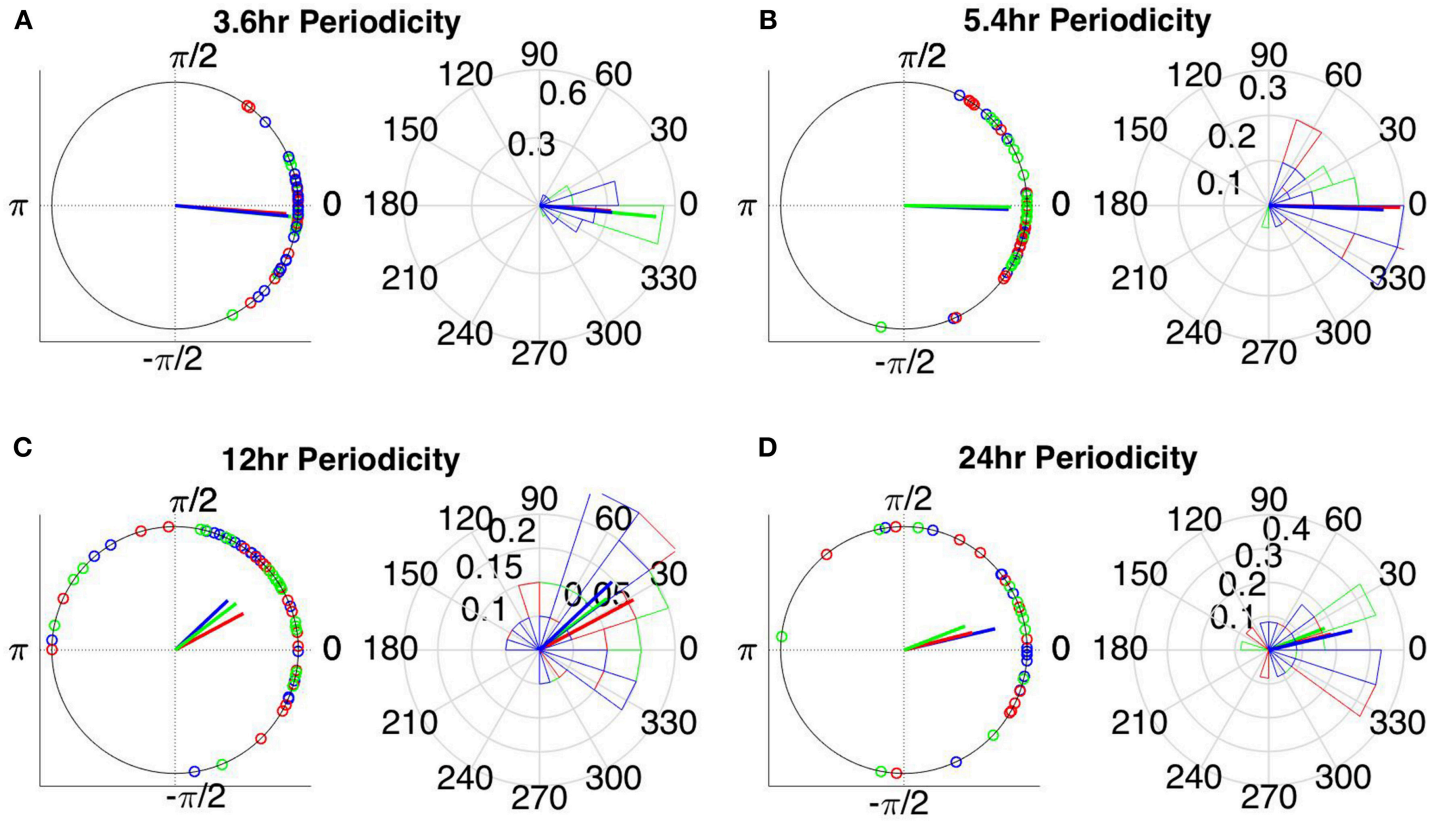
Instantaneous phases of the network average degree at seizure onset for the **(A)** 3.6 h, **(B)** 5.4 h, **(C)** 12 h, and **(D)** 24 h periodicities of all patients for WPLI. The left panels present the unit circle and the phases as points for all seizures from all patients. The right panels show the angular histogram of the distribution as well as the corresponding probability values. Blue, common reference (Cz); green, average reference; red, bipolar reference.

The instantaneous phases of the main identified periodicities (mean across patients: 3.6, 5.4, 12, and 24 h) at seizure onset are shown in Figures 9, 10 respectively. These were obtained from all seizures from nine patients (i.e., except Patient 6) for the correlation measures that were less affected by reference choice, i.e., CorCC and WPLI (Figure 8 and Figure S9). Figures 9, 10 show the instantaneous phases for the average network degree obtained using corCC and WPLI, respectively, for all montages, and peaks. The left panels show the instantaneous phases on the unit circle, while the right panels show the corresponding angular histograms. The green, red, and blue circles indicate the instantaneous phases obtained from the average, bipolar, and common (Cz) reference, respectively. The lines of the same color indicate the direction and magnitude of the mean resultant vector. The length of this vector is a crucial quantity for the measurement of circular spread and hypothesis testing in circular statistics. The closer the vector magnitude is to one, the more concentrated the data sample is around the mean direction. The instantaneous phases of the 3.6 h (Figures 9A, 10A) and 5.4 h (Figures 9B, 10B) periodicities were more concentrated around the mean direction and more consistent across montages. On the other hand, the instantaneous phases of the 12 h (Figures 9C, 10C) and 24 h (Figures 9D, 10D) periodicities were found to be less concentrated around the mean. Overall, corCC yielded the highest phase concentrations for the examined periodic components.

Table 2 shows the length of the mean resultant vector R and the corresponding *p*-values (Rayleigh test, max-values in groups) for the instantaneous phases of the average degree as obtained for all correlation measures and reference choices. It is evident from the Figures 9, 10 and Table 2 that the instantaneous phases are not distributed uniformly, but seizures occur within specific phase ranges, especially for the 3.6 and 5.4 h periodicities. With regards to the slower periodicities (12 and 24 h), the phases were distributed more uniformly around the circle, yielding non-significant values in some cases (Table 2). Overall, the bipolar montage yielded the most consistent results across all correlation measures, with the corresponding *p*-values being significant in all cases (Table 2). Overall, the results of Table 2 suggest that the coupling between seizure onset and network periodicities is detected for almost all combinations of reference and correlation measure. corCC yielded the most significant results across all periodic components followed by WPLI, while IC resulted in strong couplings for the shorter periodicities (3.6 and 5.4 h; Table 2).

**Table 2:** The values for the mean resultant vector *R* and the *p*-values obtained with Rayleigh's test for uniform distribution of the main periodic component instantaneous phases at seizure onset for all correlation measures, montages, and patients.

**Correlation measure and reference choice**		**R**	***p*-value**	**R**	***p*-value**	**R**	***p*-value**	**R**	***p*-value**
		**3.6 h**		**5.4 h**		**12 h**		**24 h**	
CC	Average	0.82	0.004	0.86	0.001	0.59	0.15	0.29	0.9
	Bipolar	0.97	2.5e-5	0.97	2.4e-5	0.85	4.3e-4	0.72	0.02
	Common	0.95	2.6e-5	0.94	3.4e-5	0.73	0.02	0.73	0.03
corCC	Average	0.97	2.4e-5	0.96	2.2e-5	0.85	0.006	0.85	0.007
	Bipolar	0.92	7.9e-5	0.91	8.9e-4	0.87	0.005	0.77	0.01
	Common	0.96	2.6e-5	0.96	2.6e-5	0.96	6.8e-4	0.9	0.004
COH	Average	0.95	1.8e-5	0.91	1.7e-5	0.75	0.09	0.88	0.003
	Bipolar	0.95	6.1e-6	0.89	0.002	0.73	0.03	0.81	0.01
	Common	0.95	2.4e-5	0.89	2.2e-4	0.65	0.15	0.51	0.6
IC	Average	0.99	2.3e-5	0.98	2.6e-5	0.91	4.1e-4	0.84	0.02
	Bipolar	0.98	2.5e-5	0.97	2.6e-5	0.88	1.4e-4	0.87	0.006
	Common	0.97	2.6e-5	0.98	2.5e-5	0.82	9.6e-4	0.69	0.09
PLI	Average	0.94	4.11e-5	0.88	8.7e-4	0.47	0.5	0.68	0.2
	Bipolar	0.95	2.6e-5	0.90	1.6e-4	0.70	0.02	0.77	0.01
	Common	0.95	2.6e-5	0.94	2.5e-5	0.66	0.3	0.83	0.01
WPLI	Average	0.95	5.2e-5	0.87	0.002	0.72	0.5	0.73	0.8
	Bipolar	0.90	2.1e-4	0.83	0.003	0.73	0.03	0.70	0.02
	Common	0.92	1.4e-4	0.85	0.002	0.79	0.01	0.86	0.009

*The p-values that are larger than 0.05, suggesting that the instantaneous phases are distributed uniformly around the circle, are indicated in red. CC, cross-correlation; corCC, corrected cross-correlation; COH, coherence; IC, imaginary coherence; PLI, phase lag index; and WPLI, weighted phase lag index*.

## Discussion

Complex network analysis has recently emerged as a promising approach for studying brain dynamics and particularly functional connectivity. However, network analyses based on signal correlations of scalp EEG recordings are affected by the choice of reference electrode (montage), as well as volume conduction and more generally the fact that common signal is picked up by different electrodes leading to spurious correlations at zero lag. In this work, we investigated the effects of reference choice and volume conduction on the long-term properties of functional brain networks obtained from scalp EEG measurements using long duration data (between 22 and 94 h) from patients with epilepsy, extending our previous studies (Christodoulakis et al., 2013; Anastasiadou et al., 2016; Mitsis et al., 2018). To do so, we examined six bivariate signal correlation measures—CC, corCC, COH, IC, PLI, and WPLI, as well as three montages—common (Cz), average and bipolar. We quantified the long-term brain network properties using three widely used graph theoretic measures: average degree, efficiency, and clustering coefficient (Rubinov and Sporns, 2010).

Overall, the results obtained using the examined correlation measures and montages revealed consistent periodicities over different time scales in the obtained brain network properties, in agreement with (Anastasiadou et al., 2016; Mitsis et al., 2018). Specifically, in these papers the presence of a main 24 h circadian periodicity as well as periodicities around 3.6, 5.4, and 12 h (harmonically related to the 24 h periodicity on a subject-specific basis) were revealed for both network summative properties and topology (Mitsis et al., 2018). Here, we have extended the results by systematically examining the effects of correlation measure and montage choice. While the locations of the main periodicities were found to be overall consistent across these choices (Figure 8 and Figures S9, S10), it was found that the choice of reference and correlation measure may have pronounced effects on the results (Figures 2–7). Specifically, the average reference was found to yield the most pronounced differences compared to the other montages. This includes a reversal of the characteristics of the main 24 h periodicity (higher average degree, global efficiency and clustering coefficient during sleep; Figures 2, 4–6 and Figures S2, S4) as well as more variability with regards to the identified frequency peaks for a subset of the employed correlation measures (Figures S9,S10).

Ideally, the EEG referencing should be performed with respect to a reference electrode with zero voltage values. However, in practice the voltage values of the reference electrode are never zero. In addition to these reference effects, in practice the data and inferences from it are also affected by volume conduction, i.e., the fact that two or more sensors may instantaneously pick up a signal from the same source. Therefore, in practice, both volume conduction and reference electrode effects will occur inevitably. This is particularly true in the clinical setting examined in the present paper, where we had a standard 10–20 setup with a low number of electrodes. However, in such a setting of limited electrode numbers and limited head electrode coverage in an empirical dataset, is not possible to directly address the reference electrode and volume conduction effects. Therefore, the effects of volume conduction were *indirectly* assessed by using measures that are *differentially* sensitive to the influence of volume conduction. Both volume conduction and common (non-zero) reference effects can result in artifactual zero-time lag correlations. Various measures/correlation metrics have been proposed that are relatively insensitive to such zero-lag correlations including corCC, IC, PLI, and WPLI. Such measures (largely) account for volume conduction effects, which are necessarily zero-time lag but also for effects of non-zero and/or common reference.

Of the six correlation measures that we investigated, CC, and COH were affected by the choice of recording reference (montage) to the greatest degree, followed by IC and PLI. This was expected overall, as CC and COH are known to be influenced by zero-lag correlations (Nunez and Srinivasan, 2006). It should be noted that zero-lag correlations could be due to both artifactual (volume conduction/reference effects) and true correlations, whereas non-zero lag correlations are more likely to reflect true correlations of underlying sources (Eggermont and Smith, 1996; Stam et al., 2007). Thus, by quantifying correlations by measures that are less sensitive to volume conduction and active reference effects, one accepts the risk of missing functionally meaningful correlations at zero-lag, but at the same time, the most frequent artifacts for misinterpretation of correlations are very much reduced (Stam et al., 2007). CorCC and WPLI were the least affected correlation measures, as all three montages yielded similar network measure patterns (Figures 3, 6 and Figures S3, S5–S7). In addition to the aforementioned differences related to periodic peaks and reversal of the main circadian pattern, the average reference yielded higher connectivity (as reflected on the average degree; Figures 2–6) compared to the other two montages for the same threshold value.

In our previous work, we have also assessed the effect of reference and correlation measure choice using relatively short data records (30 min) before and after seizure onset (Christodoulakis et al., 2014). Our results demonstrated that the graphs constructed with CC and COH were affected by volume conduction and montage more markedly; however, they exhibited a similar trend—decreasing connectivity at seizure onset, as well as during the ictal and early postictal periods, increasing again several minutes after the seizure has ended—with all the aforementioned measures accounting for volume conduction (corCC, PLI, WPLI) except IC. In particular, networks constructed using CC yielded a clearer discrimination between the pre-ictal and ictal periods than the measures less sensitive to volume conduction such as the PLI and IC. Thus, somewhat paradoxically, although removing the effects of volume conduction allows for a more accurate reconstruction of the true underlying networks this may come at the cost of discrimination ability with respect to brain state.

The average reference produces a good approximation of the reference-free potentials, given sufficient electrode coverage of the head (Nunez and Srinivasan, 2006; Nunez, 2011). However, the assumption underlying the average reference montage only holds for spherical volume conductors (Yao, 2017). As a standard 10–20 electrode system was used here, the average reference montage is likely to provide a poor approximation of the reference-free potentials (Nunez and Srinivasan, 2006, p. 295) as both the low number of electrodes and limited head coverage errors (only the upper part of the head was sampled) come into play. This is supported by our results, which suggest that using the average reference may result in considerable common signal being subtracted from each electrode, introducing artifactual correlation at zero-time lags. On the other hand, the bipolar montage yields better estimates of the local gradient of the potential along the scalp surface than a fixed reference at a remote distance. This increases sensitivity and spatial resolution for superficial generators but reduces the sensitivity to distant sources (Nunez and Srinivasan, 2006). Also, as we are assessing connectivity in the present study, the bipolar montage is a reasonable choice as it provides an estimate of local brain dynamics. When the effects of zero-time lag correlations are removed, for instance using corCC (Figure 3) or WPLI (Figure 7), the average reference montage results exhibited qualitatively similar trends to the bipolar [and common (Cz)] montage data. This supports the idea that there is possibly a remaining large common component in the average that is instantaneously subtracted from each channel.

The REST (reference electrode standardization technique) referencing method has recently been proposed to yield approximately reference-free potentials (Yao, 2001, 2017; Yao et al., 2005; Marzetti et al., 2007; Qin et al., 2010; Xu et al., 2014; Chella et al., 2016; Dong et al., 2017) and has been shown in simulations to outperform the average reference montage (Qin et al., 2010; Nunez, 2011). Ideally, the REST method is implemented by recording electrode positions and using individual head models but it can also be implemented using an average head template mode. A recent simulation study has suggested that REST outperforms average referencing even for a limited number of electrodes (Hu et al., 2018). In our experimental setting, due to that electrode positions were not recorded and the subsequent lack of an individual head model, as well as to the limited electrode density and coverage, which are typical in clinical settings, we did not implement REST. Also, we did not consider linked-ears or linked-mastoids referencing (physical or mathematical), as this approach has limited theoretical basis and may yield biased estimates of reference-free potentials (Nunez and Srinivasan, 2006).

Our results agree with previous studies (Nunez et al., 1997; Stam et al., 2007; Vinck et al., 2011; Peraza et al., 2012; Christodoulakis et al., 2014) in that corCC and WPLI were found to be less affected by reference choice. To our knowledge, our study is the first that demonstrates this for long-duration properties of the scalp EEG-based functional brain networks. Also, our results overall agree with previous studies applying graph-theoretic measures to intracranial recordings. Specifically, Kuhnert et al. (2010) recorded intracranial EEG data and constructed functional brain networks using mean phase coherence. They showed that functional brain networks change periodically over time, with prominent cycle around 24 h, and that seizures influence the brain networks significantly less compared to daily rhythms. In 2017, Geier and Lehnertz investigated the temporal and spatial variability of the importance regions in evolving epileptic brain networks and showed that the importance of brain regions fluctuates over time, with these fluctuations being mostly attributed to processes acting on timescales of hours to days.

We also assessed the effect of reference choice and correlation measure on the correlation strength between the instantaneous phase of the functional network periodicities in network properties (3.6, 5.4 and 12 h) with seizure onset revealed in our previous work (Anastasiadou et al., 2016; Mitsis et al., 2018). Importantly, in these studies we showed that connectivity-based markers are a more specific marker of seizure onset, as the couplings between long-term periodic components and seizure onset were not found to be present for the scalp EEG signals (Mitsis et al., 2018). The instantaneous phase of these periodicities (as obtained from the average degree time course) over different time scales was found to be correlated with seizure onset (Figures 9, 10 and Table 2) for almost all combinations of correlation measure and montage choices and that, overall, corCC, WPLI, and IC yielded the strongest coupling strengths (Table 2). In all cases, the instantaneous phase of the 3.6 and 5.4 h periodic components were more concentrated around the mean direction as compared to the 12 and 24 h components (Figures 9C,D, 10C,D), in agreement with (Anastasiadou et al., 2016; Mitsis et al., 2018).

In the present paper, we assumed that the period of the identified network-related periodic components is constant over time. In principle, these periods may change over longer time periods (multiple days to weeks to even months). However, to observe such non-stationarities, particularly for the slower (e.g., circadian periodicities) would require very long duration data. We are aware of only one study of this kind that used intracranial data collected over weeks/months using an implantable deep brain stimulation system, where it was shown that subject-specific multi-dien rhythms in the intracranial EEG signal properties were correlated to seizures (Baud et al., 2018). The authors of that paper did not examine network properties as brain coverage was more limited. However, as the main circadian periodicity remains approximately constant over time (around 24 h) and the shorter periodicities that were found to be correlated to seizure onset hereby were mostly harmonics of the circadian periodicity, it is expected that even if time-frequency analysis is implemented, the results would not change substantially.

In conclusion, the present study suggests that the choice of reference may considerably affect the estimated long-term properties of graph theoretic analysis of scalp EEG functional brain networks and that, for the relatively low number of electrodes examined hereby, the bipolar montage yielded the most consistent results, while corCC and WPLI were the correlation measures that were found to be least affected by reference choice. Therefore, using a bipolar montage combined with one of these correlation measures (corCC and WPLI) in similar studies may lead to a better understanding of long-term functional connectivity, as well as improved seizure prediction/detection algorithms that take into account the instantaneous phase of the underlying network periodicities.

## Author Contributions

GM, AH, and MA conceived and designed the study. EP and SP performed the experiments. MA, MC, GM, AH, EP, and SP analyzed the data. MA, GM, MC, and AH wrote the paper.

### Conflict of Interest Statement

The authors declare that the research was conducted in the absence of any commercial or financial relationships that could be construed as a potential conflict of interest.

## Supplementary Material

Figure S1 Average degree of the functional brain network of Patient 4 using cross-correlation and the bipolar montage, constructed using multivariate phase randomization surrogate data (blue line), and thresholding as in Figure 2 (red line; threshold value: 0.65). The surrogate method yielded more densely connected networks. While the temporal patterns are similar, the circadian periodicity is less clear for the surrogate-obtained results.

Figure S2 Efficiency of the functional brain networks of Patient 4 as a function of time, using cross correlation for assessing pairwise correlations. The vertical dashed line indicates seizure onset and the gray bars indicate sleep intervals. Cross correlation yields an opposite 24 h periodic pattern when using the average reference. However, we can clearly observe a periodic pattern with a main period equal to around 24 h for all montages.

Figure S3 Efficiency of the functional brain networks of Patient 4 as a function of time, using corrected cross correlation for assessing pairwise correlations. The vertical dashed line indicates seizure onset and the gray bars indicate sleep intervals. In contrast to cross correlation (Figure S2), corrected cross correlation yields the same 24 h periodic pattern for all montages.

Figure S4 Clustering coefficient of the functional brain networks of Patient 4 as a function of time using cross correlation for assessing pairwise correlations. The vertical dashed line indicates seizure onset and the gray bars indicate sleep intervals. Cross correlation yields an opposite 24 h periodic pattern when using the average reference, similarly in efficiency (Figure S1). However, we can clearly observe a periodic pattern with a main period equal to around 24 h for all montages.

Figure S5 Clustering coefficient of the functional brain networks of Patient 4 as a function of time, using corrected cross correlation for assessing pairwise correlations. The vertical dashed line indicates seizure onset and the gray bars indicate sleep intervals. In contrast to cross correlation (Figure S3), corrected cross correlation yields the same 24 h periodic pattern for all montages similarly in efficiency (Figure S2).

Figure S6 Efficiency of the functional brain networks of Patient 4 as a function of time (broadband signal), using WPLI for assessing pairwise correlations. The vertical dashed line indicates seizure onset and the gray bars indicate sleep intervals. Similarly, to corrected cross correlation (Figures S2, S3), WPLI yields the same 24 h periodic pattern for all montages.

Figure S7 Clustering coefficient of the functional brain networks of Patient 4 as a function of time (broadband signal), using WPLI for assessing pairwise correlations. The vertical dashed line indicates seizure onset and the gray bars indicate sleep intervals. Similarly to corrected cross correlation (Figures S2, S3), WPLI yields the same 24 h periodic pattern for all montages as in Figure S5 with efficiency.

Figure S8 Lomb-Scargle periodogram of the average degree of the functional brain network of Patient 4 using cross-correlation, corrected cross-correlation, coherence, imaginary coherence, PLI, and WPLI for all montages. The dotted horizontal lines denote the statistical significance level (*p* = 0.05). The arrows in the inset figures denote the periods around 3.6 and 5.4 h, which were identified across all subjects along with the peaks around 12 and 24 h. Corrected cross correlation and WPLI were affected less by reference choice.

Figure S9 Lomb-Scargle periodogram of the average degree of the functional brain network of Patient 4 obtained using cross-correlation and bipolar montage, whereby binarization was performed using surrogate data. As suggested in Figure S2, the circadian periodicity is less pronounced compared to networks obtained using thresholding.

Figure S10 Lomb-Scargle periodogram of the average degree of the functional brain network of Patient 6 (psychogenic seizure patient) using cross-correlation, corrected cross-correlation, coherence, imaginary coherence, PLI, and WPLI for common reference (blue line) and bipolar montage (red line). The dotted horizontal lines denote the statistical significance level (*p* = 0.05). The arrows in the inset figures denote the periods around 3.6 and 5.4 h, which were identified across all subjects along with the peaks around 12 and 24 h. Corrected cross correlation and WPLI were affected less by reference choice.

## References

[B1] Anastasiadou M. Hadjipapas A. Christodoulakis M. Papathanasiou E. S. Papacostas S. S. Mitsis G. D. (2016). Epileptic seizure onset correlates with long term eeg functional brain network properties^*^, in 2016 IEEE 38th Annual International Conference of the Engineering in Medicine and Biology Society (EMBC) (Orlando, FL), 2822–2825. 10.1109/EMBC.2016.7591317 28268905

[B2] Baud M. O. Kleen J. K. Mirro E. A. Andrechak J. C. King-Stephens D. Chang E. F.. (2018). Multi-day rhythms modulate seizure risk in epilepsy. Nat. Commun. 9:88. 10.1038/s41467-017-02577-y 29311566 PMC5758806

[B3] Berens P. (2009). CircStat : a MATLAB toolbox for circular statistics. J. Stat. Softw. 31, 1–21. 10.18637/jss.v031.i10

[B4] Burns S. P. Santaniello S. Yaffe R. B. Jouny C. C. Crone N. E. Bergey G. K.. (2014). Network dynamics of the brain and influence of the epileptic seizure onset zone. Proc. Natl. Acad. Sci. U.S.A. 111, E5321–5330. 10.1073/pnas.1401752111 25404339 PMC4267355

[B5] Chella F. Pizzella V. Zappasodi F. Marzetti L. (2016). Impact of the reference choice on scalp EEG connectivity estimation. J. Neural Eng. 13:36016. 10.1088/1741-2560/13/3/036016 27138114

[B6] Christodoulakis M. Hadjipapa A. Papathanasiou E. S. Anastasiadou M. Papacostas S. S. Mitsis G. D. (2013). On the effect of volume conduction on graph theoretic measures of brain networks in epilepsy, in Modern Electroencephalographic Assessment Techniques. Neuromethods, ed Sakkalis V. (New York, NY: Humana Press), 103–130. 10.1007/7657_2013_65

[B7] Christodoulakis M. Hadjipapas A. Papathanasiou E. S. Anastasiadou M. Papacostas S. Mitsis G. (2014). On the effect of volume conduction on graph theoretic measures of brain networks in epilepsy, in Modern Electroencephalographic Assessment Techniques. Neuromethods, ed Sakkalis V. (New York, NY: Springer), 103–30.

[B8] Dong L. Li F. Liu Q. Wen X. Lai Y. Xu P.. (2017). MATLAB toolboxes for reference electrode standardization technique (REST) of scalp EEG. Front Neurosci. 11:601. 10.3389/fnins.2017.00601 29163006 PMC5670162

[B9] Eggermont J. J. Smith G. M. (1996). Neural connectivity only accounts for a small part of neural correlation in auditory cortex. Exp. Brain Res. 110, 379–91. 8871097 10.1007/BF00229138

[B10] Fisher N. I. (1993). Statistical Analysis of Circular Data. Cambridge: Cambridge University Press.

[B11] Geier C. Bialonski S. Elger C. E. Lehnertz K. (2015). How important is the seizure onset zone for seizure dynamics'. Seizure 25, 160–166. 10.1016/j.seizure.2014.10.013 25468511

[B12] Guevara R. Velazquez J. L. Nenadovic V. Wennberg R. Senjanovic G. Dominguez L. G. (2005). Phase synchronization measurements using electroencephalographic recordings: what can we really say about neuronal synchrony? Neuroinformatics 3, 301–314. 10.1385/NI:3:4:301 16284413

[B13] Haufe S. Tomioka R. Nolte G. Müller K. R. Kawanabe M. (2010). Modeling sparse connectivity between underlying brain sources for EEG/MEG. IEEE Trans. Bio-Med. Eng. 57, 1954–1963. 10.1109/TBME.2010.2046325 20483681

[B14] Hu S. Lai Y. Valdés-Sosa P. A. Brings-Vega M. L. Yao D. (2018). How do the reference montage and electrodes setup affect the measured scalp EEG potentials? J. Neural Eng. 15:026013. 10.1088/1741-2552/aaa13f 29368697

[B15] Klingspor M. (2015). Hilbert Transform : Mathematical Theory and Applications to Signal Processing. Faculty of Science and Engineering, Linköping University.

[B16] Kramer M. A. Eden U. T. Lepage K. Q. Kolaczyk E. D. Bianchi M. T. Cash S. S. (2011). Emergence of persistent networks in long-term intracranial {EEG} recordings. J. Neurosci. 31, 15757–15767. 10.1523/JNEUROSCI.2287-11.2011 22049419 PMC3226835

[B17] Kramer M. A. Kolaczyk E. D. Kirsch H. E. (2008). Emergent network topology at seizure onset in humans. Epilepsy Res. 79, 173–186. 10.1016/j.eplepsyres.2008.02.002 18359200

[B18] Kuhnert M. T. Elger C. E. Lehnertz K. (2010). Long-term variability of global statistical properties of epileptic brain networks. Chaos 20:43126. 10.1063/1.3504998 21198096

[B19] Latora V. Marchiori M. (2001). Efficient behavior of small-world networks. Phys. Rev. Lett. 87:198701. 10.1103/PhysRevLett.87.198701 11690461

[B20] Lehnertz K. Ansmann G. Bialonski S. Dickten H. Geier C. Porz S. (2014). Evolving networks in the human epileptic brain. Physica D 267, 7–15. 10.1016/j.physd.2013.06.009

[B21] Marzetti L. Nolte G. Perrucci M. G. Romani G. L. Del Gratta C. (2007). The use of standardized infinity reference in EEG coherency studies. NeuroImage 36, 48–63. 10.1016/j.neuroimage.2007.02.034 17418592

[B22] Mitsis G. D. Anastasiadou M. N. Christodoulakis M. Papathanasiou E. S. Papacostas S. S. Hadjipapas A. (2018). Multi-scale periodicities in the functional brain networks of patients with epilepsy and their effect on seizure detection. bioRxiv[preprint]. bioRxiv:221036 10.1101/221036

[B23] Nevado A. Hadjipapas A. Kinsey K. Moratti S. Barnes G. R. Holliday I. E.. (2012). Estimation of functional connectivity from electromagnetic signals and the amount of empirical data required. Neurosci. Lett. 513, 57–61. 10.1016/j.neulet.2012.02.007 22329975

[B24] Nicolaou N. Nasuto S. J. (2007). Automatic artefact removal from event-related potentials via clustering. J. VLSI Signal Process. Syst. Signal Image Video Technol. 48, 173–183. 10.1007/s11265-006-0011-z

[B25] Nolte G. Bai O. Wheaton L. Mari Z. Vorbach S. Hallett M. (2004). Identifying true brain interaction from EEG data using the imaginary part of coherency. Clin Neurophysiol. 115, 2292–2307. 10.1016/j.clinph.2004.04.029 15351371

[B26] Nunez P. L. (2011). REST: a good idea but not the gold standard. Clin. Neurophysiol. 121, 2177–2180. 10.1016/j.clinph.2010.04.029 PMC2967671 20554245

[B27] Nunez P. L. Silberstein R. B. Shi Z. Carpenter M. R. Srinivasan R. Tucker D. M.. (1999). EEG coherency II: experimental comparisons of multiple measures. Clin. Neurophysiol. 110, 469–486. 10.1016/S1388-2457(98)00043-1 10363771

[B28] Nunez P. L. Srinivasan R. (2006). Electric Fields of the Brain: The Neurophysics of EEG. New York, NY: Oxford University Press.

[B29] Nunez P. L. Srinivasan R. Westdorp A. F. Wijesinghe R. S. Tucker D. M. Silberstein R. B.. (1997). EEG coherency. I: statistics, reference electrode, volume conduction, Laplacians, cortical imaging, and interpretation at multiple scales. Electroencephalogr. Clin. Neurophysiol. 103, 499–515. 10.1016/S0013-4694(97)00066-7 9402881

[B30] Peraza L. R. Asghar A. U. Green G. Halliday D. M. (2012). Volume conduction effects in brain network inference from electroencephalographic recordings using phase lag index. J. Neurosci. Methods 207, 189–199. 10.1016/j.jneumeth.2012.04.007 22546477

[B31] Pereda E. Quiroga R. Q. Bhattacharya J. (2005). Nonlinear multivariate analysis of neurophysiological signals. Prog. Neurobiol. 77, 1–37. 10.1016/j.pneurobio.2005.10.003 16289760

[B32] Qin Y. Xu P. Yao D. (2010). A comparative study of different references for EEG default mode network: the use of the infinity reference. Clin. Neurophysiol. 121, 1981–1991. 10.1016/j.clinph.2010.03.056 20547470

[B33] Rubinov M. Sporns O. (2010). Complex network measures of brain connectivity: uses and interpretations. NeuroImage 52, 1059–1069. 10.1016/j.neuroimage.2009.10.003 19819337

[B34] Scargle J. D. (1982). Studies in astronomical time series analysis. II. Statistical aspects of spectral analysis of unevenly spaced data. Astrophys. J. 263, 835–853. 10.1086/160554

[B35] Stam C. J. Nolte G. Daffertshofer A. (2007). Phase lag index: assessment of functional connectivity from multi channel EEG and MEG with diminished bias from common sources. Hum. Brain Mapp. 28, 1178–1193. 10.1002/hbm.20346 17266107 PMC6871367

[B36] Thatcher R. W. (2012). Coherence, phase differences, phase shift, and phase lock in EEG/ERP analyses. Dev. Neuropsychol. 37, 476–496. 10.1080/87565641.2011.619241 22889341

[B37] Theiler J. Eubank S. Longtin A. Galdrikian B. Farmer J. D. (1992). Testing for nonlinearity in time series: the method of surrogate data. Physica D 58, 77–94. 10.1016/0167-2789(92)90102-S

[B38] Varotto G. Tassi L. Franceschetti S. Spreafico R. Panzica F. (2012). Epileptogenic networks of type II focal cortical dysplasia: a stereo-EEG study. NeuroImage 61, 591–598. 10.1016/j.neuroimage.2012.03.090 22510255

[B39] Vinck M. Oostenveld R. van Wingerden M. Battaglia F. Pennartz C. M. (2011). An improved index of phase-synchronization for electrophysiological data in the presence of volume-conduction, noise and sample-size bias. NeuroImage 55, 1548–1565. 10.1016/j.neuroimage.2011.01.055 21276857

[B40] Watts D. J. Strogatz S. H. (1998). Collective dynamics of “small-world” networks. Nature 393, 440–442. 9623998 10.1038/30918

[B41] Wilke C. Worrell G. He B. (2011). Graph analysis of epileptogenic networks in human partial epilepsy. Epilepsia 52, 84–93. 10.1111/j.1528-1167.2010.02785.x 21126244 PMC3200119

[B42] Xu P. Xiong X. C. Xue Q. Tian Y. Peng Y. Zhang R.. (2014). Recognizing mild cognitive impairment based on network connectivity analysis of resting EEG with zero reference. Physiol. Measure. 35:1279. 10.1088/0967-3334/35/7/1279 24853724

[B43] Yao D. (2001). A method to standardize a reference of scalp EEG recordings to a point at infinity. Physiol Meas. 22, 693–711. 10.1088/0967-3334/22/4/305 11761077

[B44] Yao D. (2017). Is the surface potential integral of a dipole in a volume conductor always zero? a cloud over the average reference of EEG and ERP. Brain Topogr. 30, 161–171. 10.1007/s10548-016-0543-x 28194613 PMC5331115

[B45] Yao D. Wang L. Oostenveld R. Nielsen K. D. Arendt-Nielsen L. Chen A. C. (2005). A comparative study of different references for EEG spectral mapping: The issue of the neutral reference and the use of the infinity reference. Physiol. Measure. 26, 173–184. 10.1088/0967-3334/26/3/003 15798293

[B46] Zar J. H. (1999). Biostatistical Analysis. Upper Saddle River, NJ: Prentice Hall.

[B47] Zubler F. Gast H. Abela E. Rummel C. Hauf M. Wiest R.. (2014). Detecting functional hubs of ictogenic networks. Brain Topogr. 28, 305–317. 10.1007/s10548-014-0370-x 24846350

