# Supplementary figures and images for: Graph Theoretical Characteristics of EEG-Based Functional Brain Networks in Patients With Epilepsy: The Effect of Reference Choice and Volume Conduction
Source: Front Neurosci. 2019 Mar 20;13:221. doi: 10.3389/fnins.2019.00221 (PMC6436604; doi:10.3389/fnins.2019.00221)

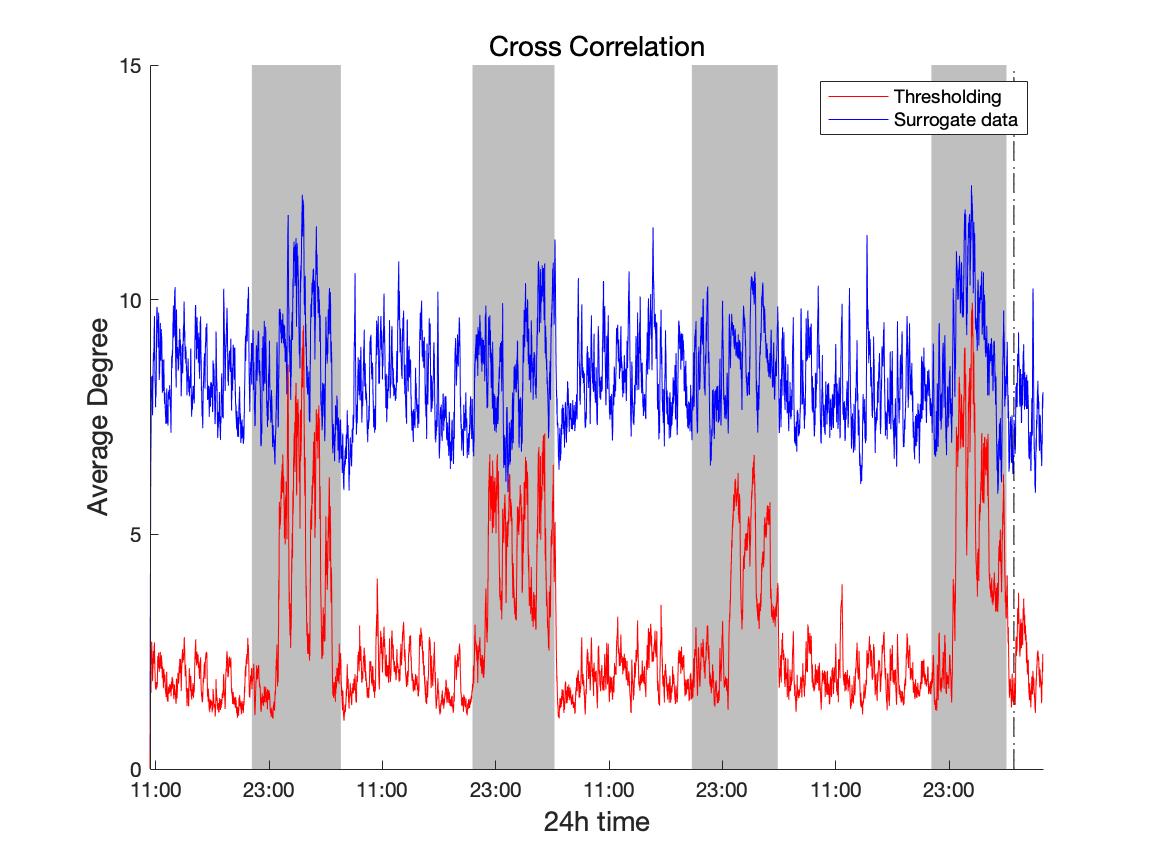

Supplement: Figure S1 — Average degree of the functional brain network of Patient 4 using cross-correlation and the bipolar montage, constructed using multivariate phase randomization surrogate data (blue line), and thresholding as in Figure 2 (red line; threshold value: 0.65). The surrogate method yielded more densely connected networks. While the temporal patterns are similar, the circadian periodicity is less clear for the surrogate-obtained results. [file Image_1.JPEG]

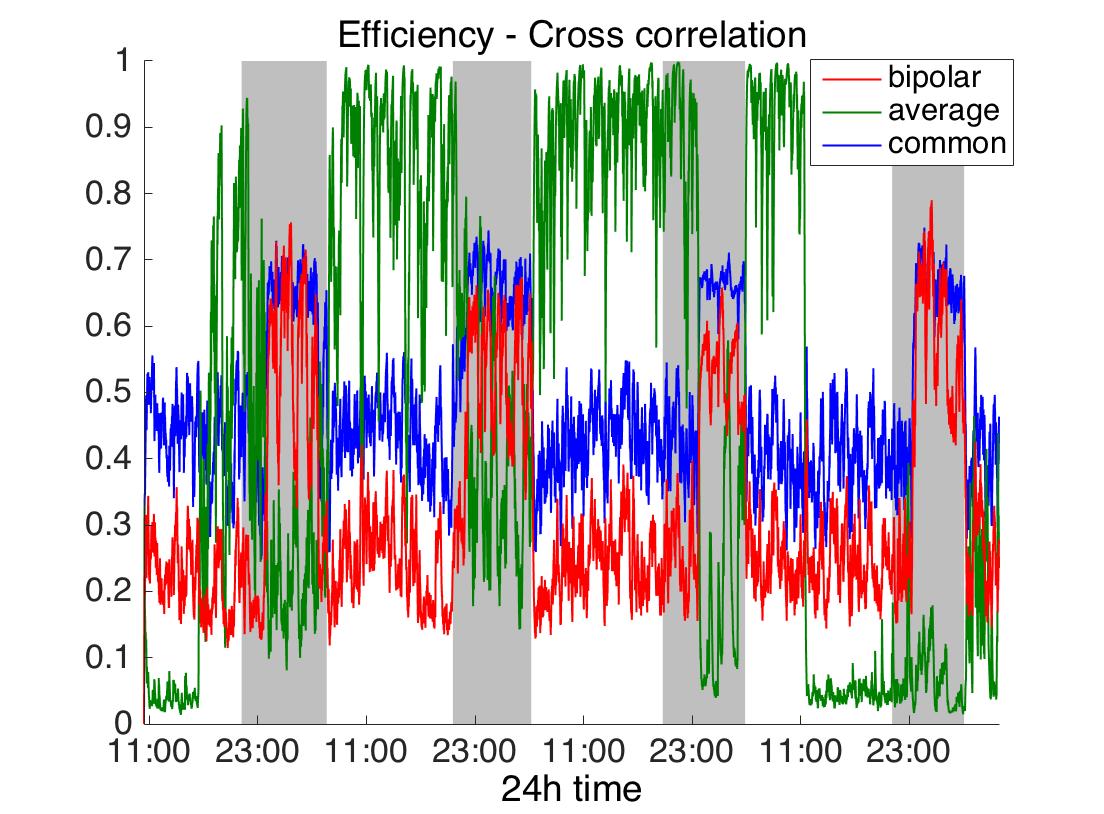

Supplement: Figure S2 — Efficiency of the functional brain networks of Patient 4 as a function of time, using cross correlation for assessing pairwise correlations. The vertical dashed line indicates seizure onset and the gray bars indicate sleep intervals. Cross correlation yields an opposite 24 h periodic pattern when using the average reference. However, we can clearly observe a periodic pattern with a main period equal to around 24 h for all montages. [file Image_2.JPEG]

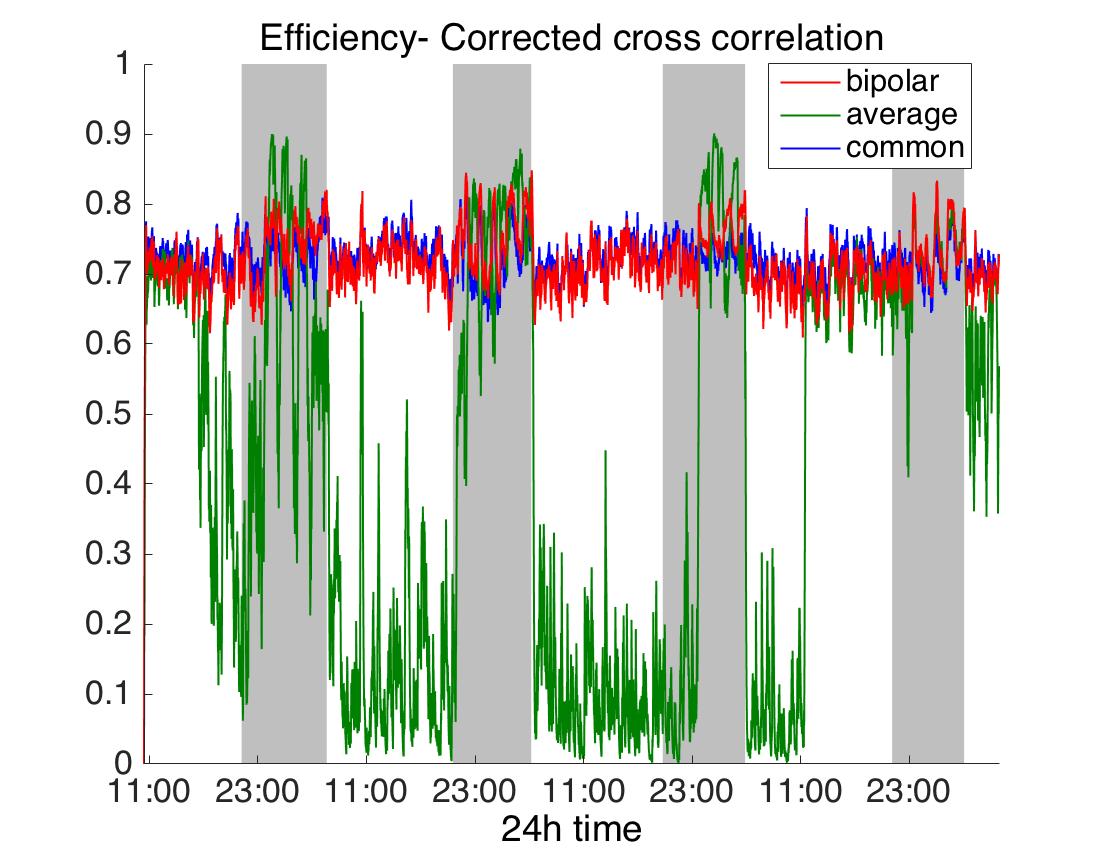

Supplement: Figure S3 — Efficiency of the functional brain networks of Patient 4 as a function of time, using corrected cross correlation for assessing pairwise correlations. The vertical dashed line indicates seizure onset and the gray bars indicate sleep intervals. In contrast to cross correlation (Figure S2), corrected cross correlation yields the same 24 h periodic pattern for all montages. [file Image_3.JPEG]

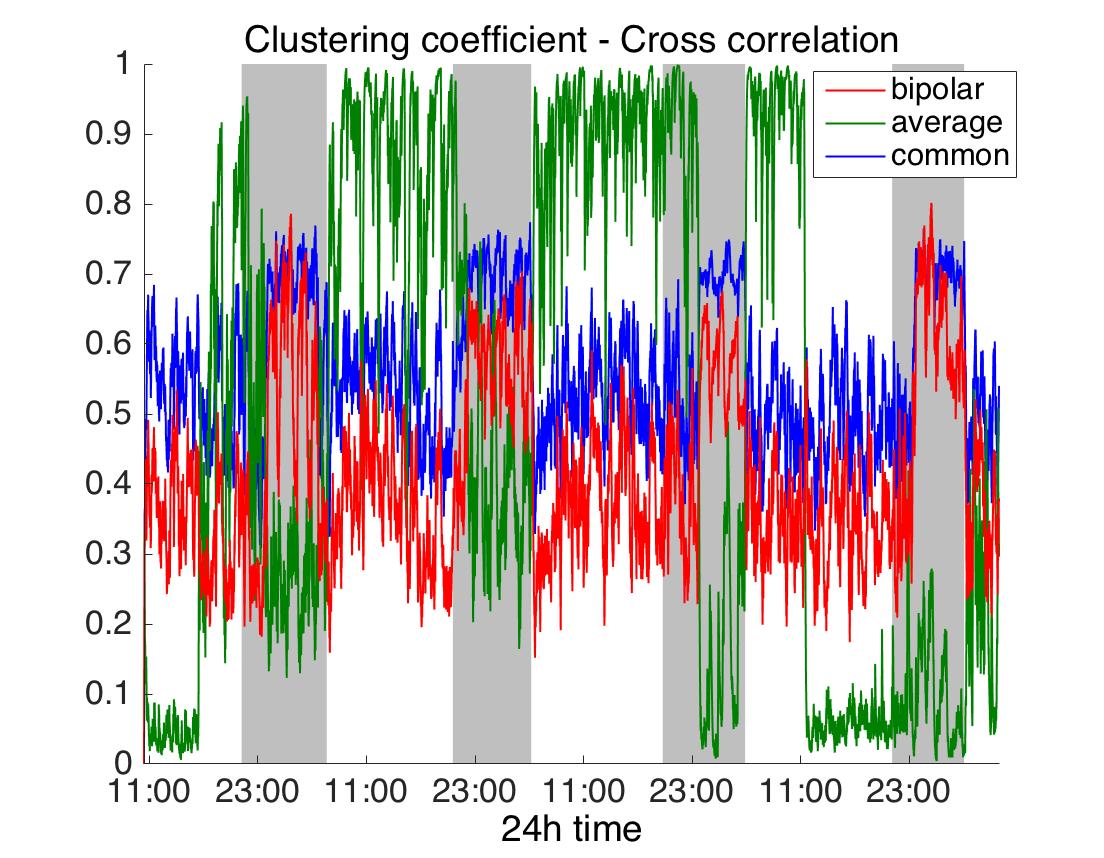

Supplement: Figure S4 — Clustering coefficient of the functional brain networks of Patient 4 as a function of time using cross correlation for assessing pairwise correlations. The vertical dashed line indicates seizure onset and the gray bars indicate sleep intervals. Cross correlation yields an opposite 24 h periodic pattern when using the average reference, similarly in efficiency (Figure S1). However, we can clearly observe a periodic pattern with a main period equal to around 24 h for all montages. [file Image_4.JPEG]

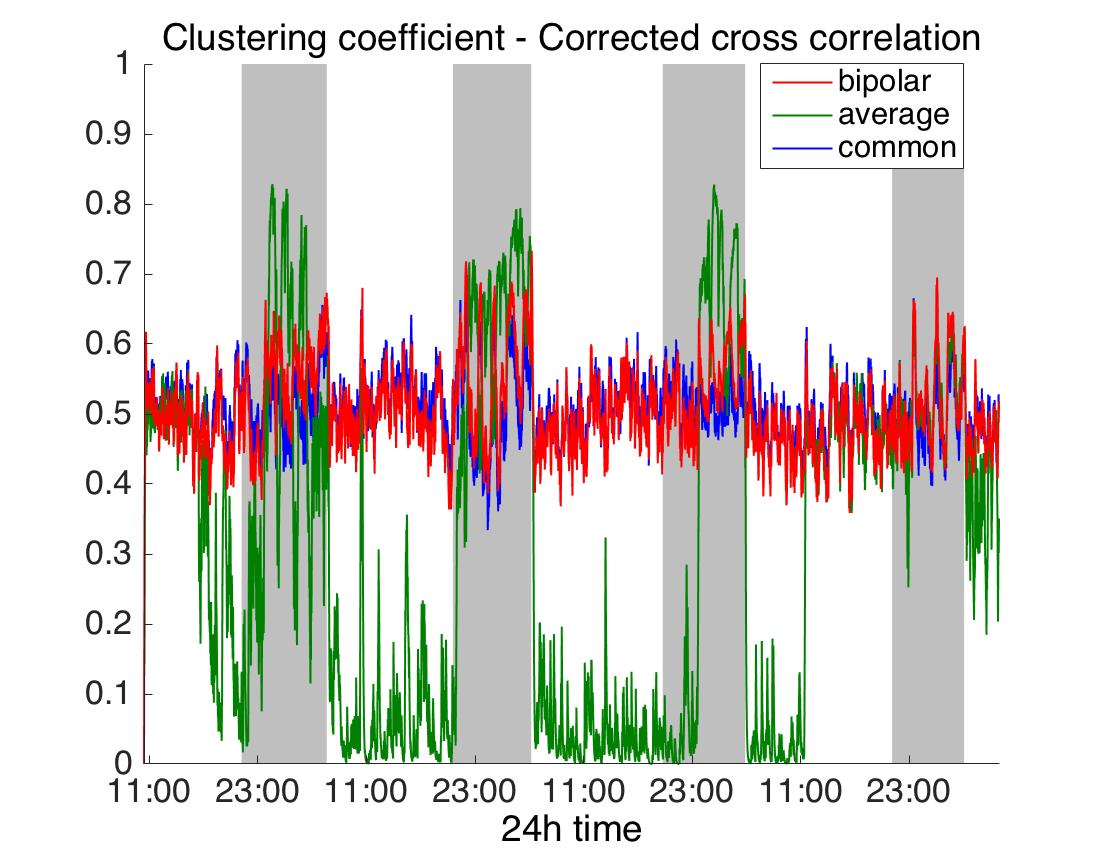

Supplement: Figure S5 — Clustering coefficient of the functional brain networks of Patient 4 as a function of time, using corrected cross correlation for assessing pairwise correlations. The vertical dashed line indicates seizure onset and the gray bars indicate sleep intervals. In contrast to cross correlation (Figure S3), corrected cross correlation yields the same 24 h periodic pattern for all montages similarly in efficiency (Figure S2). [file Image_5.JPEG]

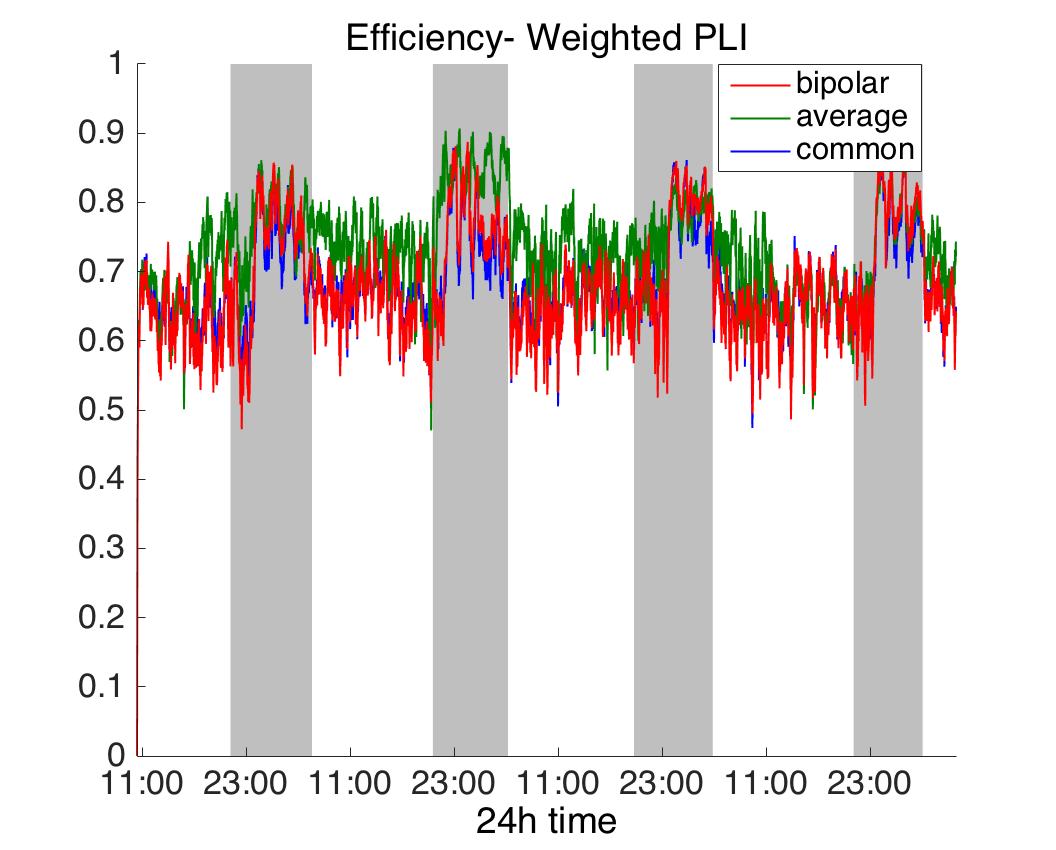

Supplement: Figure S6 — Efficiency of the functional brain networks of Patient 4 as a function of time (broadband signal), using WPLI for assessing pairwise correlations. The vertical dashed line indicates seizure onset and the gray bars indicate sleep intervals. Similarly, to corrected cross correlation (Figures S2, S3), WPLI yields the same 24 h periodic pattern for all montages. [file Image_6.JPEG]

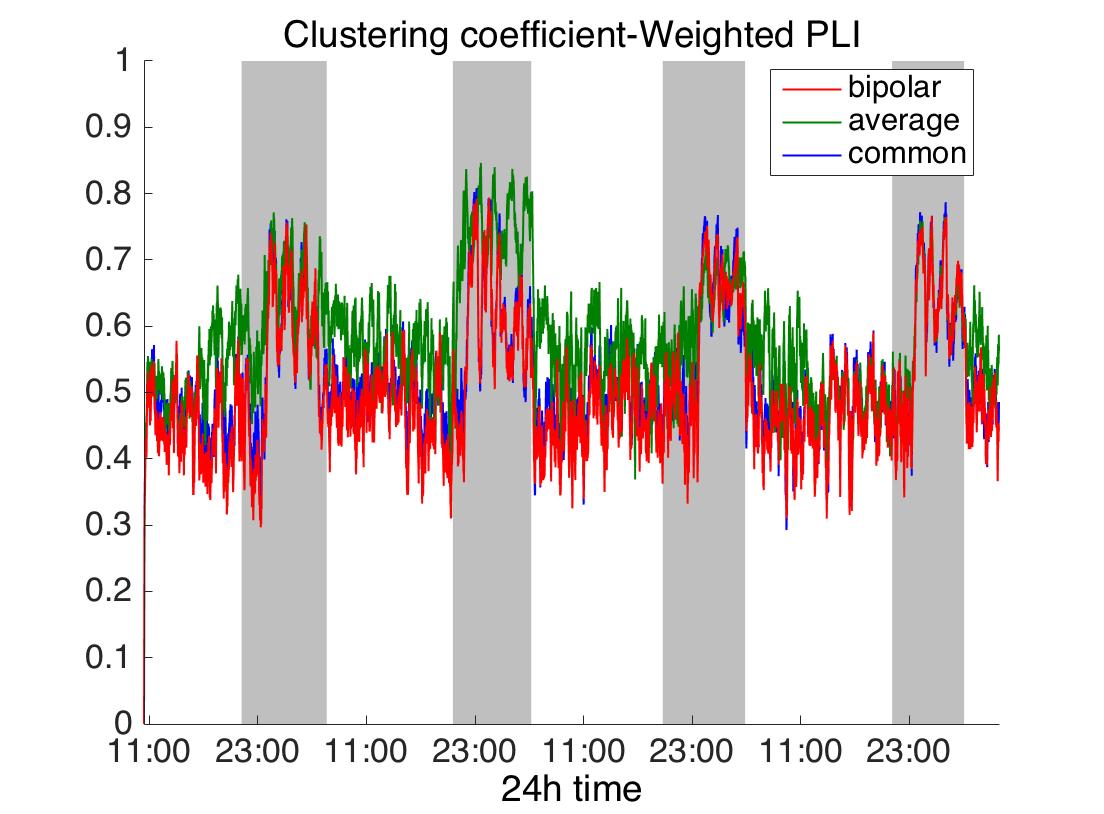

Supplement: Figure S7 — Clustering coefficient of the functional brain networks of Patient 4 as a function of time (broadband signal), using WPLI for assessing pairwise correlations. The vertical dashed line indicates seizure onset and the gray bars indicate sleep intervals. Similarly to corrected cross correlation (Figures S2, S3), WPLI yields the same 24 h periodic pattern for all montages as in Figure S5 with efficiency. [file Image_7.JPEG]

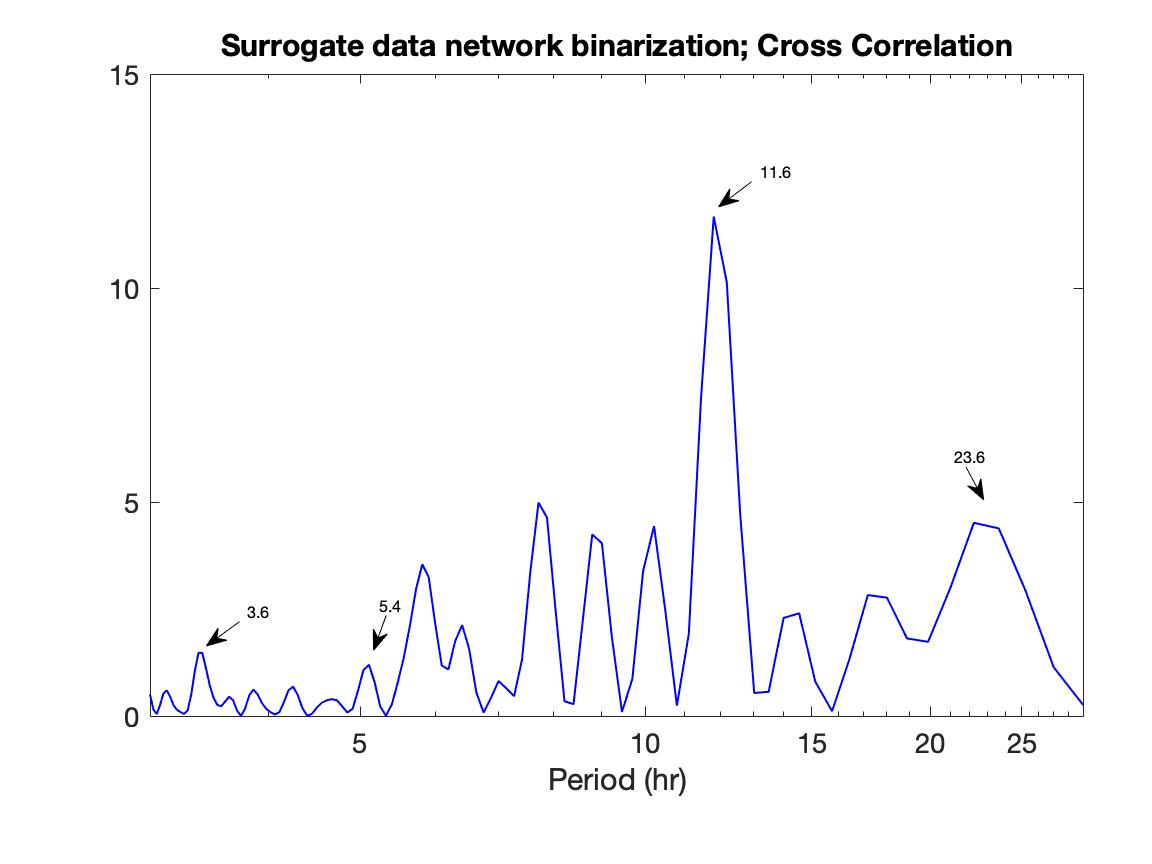

Supplement: Figure S8 — Lomb-Scargle periodogram of the average degree of the functional brain network of Patient 4 using cross-correlation, corrected cross-correlation, coherence, imaginary coherence, PLI, and WPLI for all montages. The dotted horizontal lines denote the statistical significance level (p = 0.05). The arrows in the inset figures denote the periods around 3.6 and 5.4 h, which were identified across all subjects along with the peaks around 12 and 24 h. Corrected cross correlation and WPLI were affected less by reference choice. [file Image_8.JPEG]

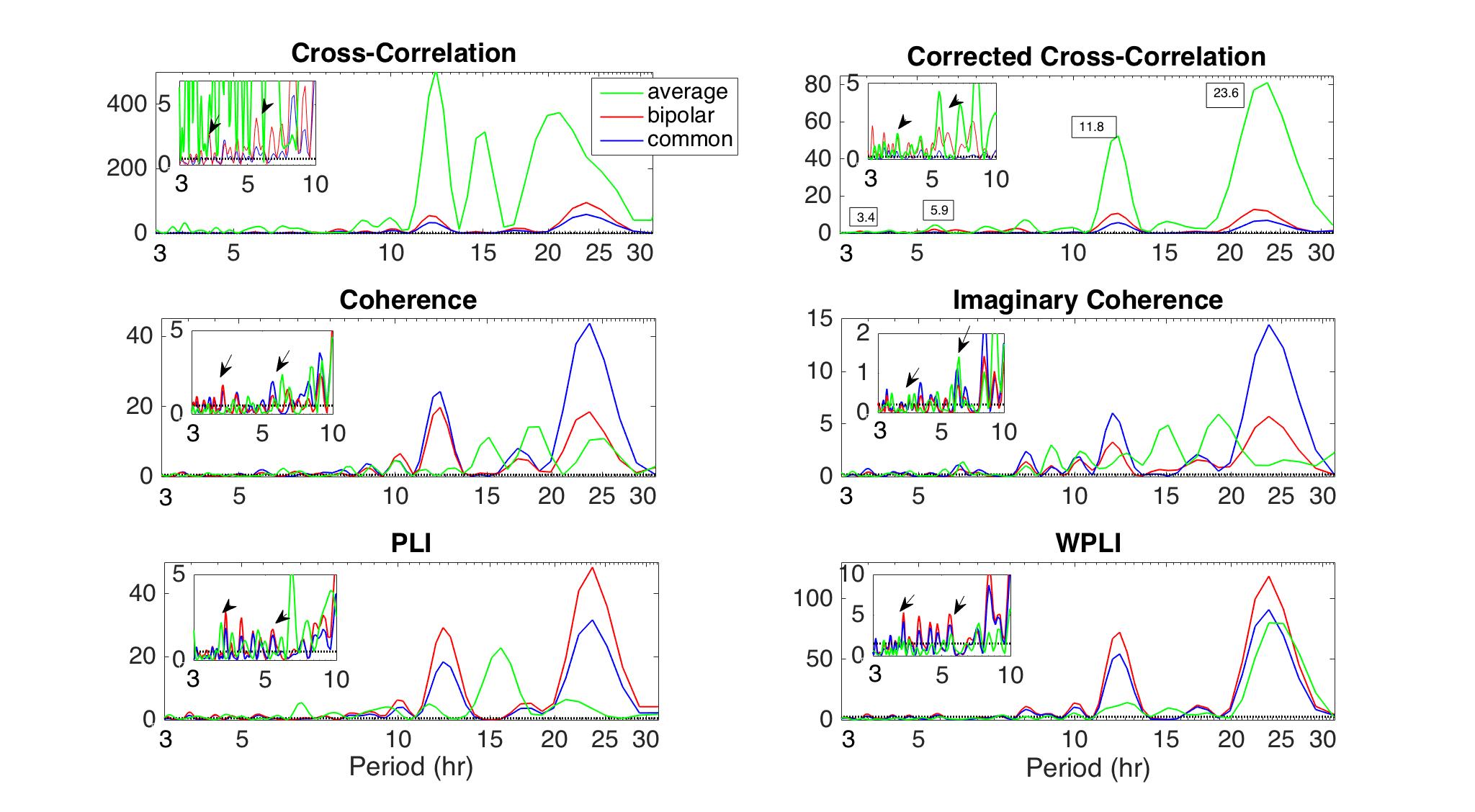

Supplement: Figure S9 — Lomb-Scargle periodogram of the average degree of the functional brain network of Patient 4 obtained using cross-correlation and bipolar montage, whereby binarization was performed using surrogate data. As suggested in Figure S2, the circadian periodicity is less pronounced compared to networks obtained using thresholding. [file Image_9.JPEG]

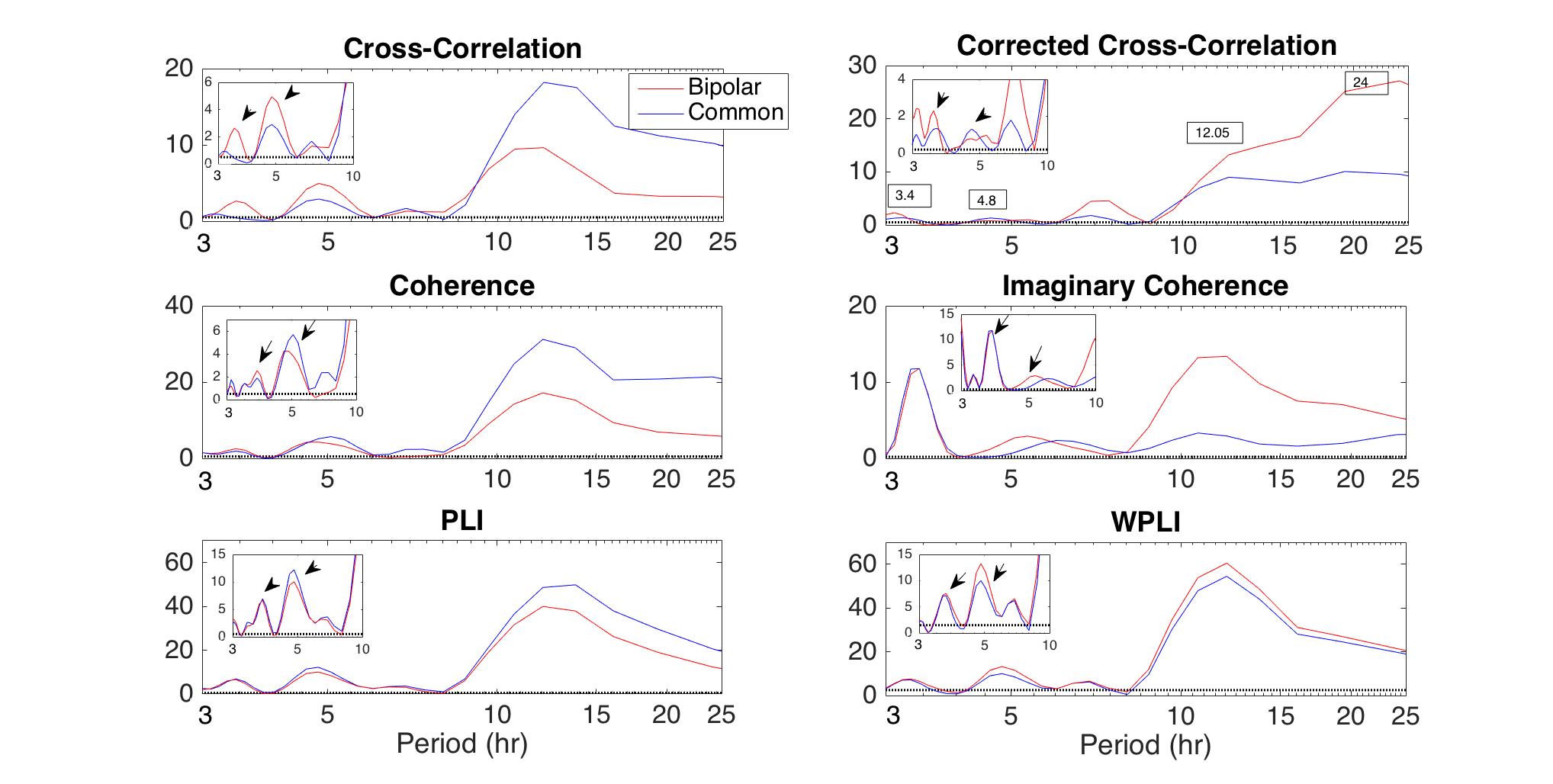

Supplement: Figure S10 — Lomb-Scargle periodogram of the average degree of the functional brain network of Patient 6 (psychogenic seizure patient) using cross-correlation, corrected cross-correlation, coherence, imaginary coherence, PLI, and WPLI for common reference (blue line) and bipolar montage (red line). The dotted horizontal lines denote the statistical significance level (p = 0.05). The arrows in the inset figures denote the periods around 3.6 and 5.4 h, which were identified across all subjects along with the peaks around 12 and 24 h. Corrected cross correlation and WPLI were affected less by reference choice. [file Image_10.JPEG]
